# Maternal early warning system (MEWS) model for predicting and reducing severe maternal outcomes in Ethiopia

**DOI:** 10.1371/journal.pone.0356105

**Published:** 2026-08-14

**Authors:** Saba Desta Tessema, Mesfin Tadese, Solomon Hailemeskel, Chaltu Takele Mule, Getaneh Dejen Tiche, Lidya Asalefew Mekonnen, Getnet Mitike Kassie

**Affiliations:** 1 Department of Midwifery, School of Nursing and Midwifery, Asrat Woldeyes Health Science Campus, Debre Berhan University, Debre Berhan, Ethiopia; 2 Department of Public Health, School of Public Health, Asrat Woldeyes Health Science Campus, Debre Berhan University, Debre Berhan, Ethiopia; 3 Department of Obstetrics and Gynecology, School of Medicine, Asrat Woldeyes Health Science Campus, Debre Berhan University, Debre Berhan, Ethiopia; 4 Department of Medicine, School of Medicine, Asrat Woldeyes Health Science Campus, Debre Berhan University, Debre Berhan, Ethiopia; 5 Senior Research advisor, International Institute for Primary Health Care – Ethiopia, Addis Ababa, Ethiopia; Clinton Health Access Initiative, UNITED STATES OF AMERICA

## Abstract

**Background:**

Maternal mortality in Ethiopia remains high, while most of these deaths are preventable. Early detection of deterioration and prompt response are essential to reduce these preventable deaths. The Maternal Early Warning System (MEWS) is a reliable clinical tool for this purpose. However, its effectiveness is underexplored and its bedside use is inconsistent. This study evaluated the MEWS model for predicting and reducing severe maternal outcomes.

**Method:**

A parallel, quasi-experimental study design was conducted among 1138 obstetric inpatients at four public hospitals of North Shewa Zone, Ethiopia. The recruitment period was from 05/05/2025–31/8/2025. The intervention group (n = 569) was monitored using the MEWS chart, which included vital signs, oxygen saturation, urine output, consciousness, and pain, and for the postpartum women; vaginal bleeding, uterine contraction, and perineal tear, were categorized as Green, Yellow, or Red. The control group (n = 569) received the standard clinical monitoring. A multivariate generalized estimating equation (GEE) model with Poisson regression was used to compare the outcomes and estimate adjusted risk ratios (aRR) with 95% confidence intervals.

**Result:**

The mean duration from admission to the first trigger was shorter by 4.7 hours (5.61 vs. 10.27 hours), trigger to physician evaluation by 22.6 minutes (49.3 vs. 71.9 minutes), and trigger to clinical intervention by 11.3 minutes (14.6 vs. 25.9 minutes) among women in the intervention group. Women also underwent fewer ultrasound scans (1.32 vs. 2.30) and had a shorter hospital stay by about 0.5 days (4.83 vs. 5.29 days). Women monitored with the MEWS chart had a 20% lower risk of severe maternal outcomes (aRR = 0.85, 95% CI: 0.73–0.99). Additionally, MEWS-monitored women were 9% more likely to be triggered for timely clinical response (aRR = 1.15, 95% CI: 1.03–1.28).

**Conclusion:**

Implementation of the MEWS was associated with earlier detection of maternal deterioration, shorter clinical response, fewer ultrasound investigations, shorter hospital stays, and lower severe maternal outcomes. Further studies with larger number of clusters are needed to evaluate the effectiveness of MEWS across different risk groups and settings.

**Trial registration:**

Pan African Clinical Trial Registry (PACTR), PACTR202506739780428, https://pactr.samrc.ac.za

## Introduction

Despite a 34% global decline in maternal mortality from 2000 to 2020, an estimated 287,000 women still died in 2020, with 95% of these deaths occurring in low-income countries, including Ethiopia [1]. In Ethiopia, the maternal mortality ratio declined from 871 per 100,000 live births in 2000–267 in 2020 [2], but this remains far above the Sustainable Development Goal target of 70 per 100,000 [3]. Beyond mortality, the World Health Organization (WHO) estimates that for every maternal death, 20–30 women experience maternal morbidity, underscoring the magnitude of the problem [4]. Nearly three-quarters of maternal deaths are attributable to hemorrhage, infections, hypertensive disorders, delivery complications, and unsafe abortion, conditions that are largely preventable with timely detection and intervention [4,5].

Delays in recognizing and managing complications remain a critical barrier to improving outcomes. Evidence from high-income countries demonstrates that the Maternal Early Warning System (MEWS), a structured bedside tool for monitoring physiological parameters, enhances early detection of deterioration and facilitates timely intervention, thereby reducing severe maternal complications and deaths [6]. MEWS systematically tracks indicators such as blood pressure, heart rate, respiratory rate, temperature, oxygen saturation, urine output, consciousness, pain, and postpartum-specific parameters, including vaginal bleeding, uterine contraction, and perineal status [7]. These are categorized as “Green,” “Yellow,” or “Red,” providing clear escalation triggers for clinical response [8].

Globally, studies provide strong evidence of MEWS effectiveness. In Finland, the system showed high sensitivity in detecting leading causes of maternal mortality, such as preeclampsia, sepsis, and postpartum hemorrhage [7]. In Indonesia, it improved the frequency of patient monitoring and facilitated timely interventions [9], while in Nigeria, it enhanced early detection of deterioration and supported healthcare providers in managing workload [6]. Studies in Spain [10], China [11], and the UK [12] similarly demonstrated that MEWS trigger/activation was strongly associated with severe maternal complications. A systematic review concluded that MEWS improves routine monitoring, reduces delays in response to abnormal findings, and can lower the severity of maternal morbidity [13]. However, evidence from low-resource settings remains limited, and findings from some contexts highlight challenges in implementation fidelity [14].

Despite Ethiopia’s progress in reducing maternal mortality, preventable maternal morbidity and mortality remain high, compounded by suboptimal quality of care and reliance on reactive monitoring systems [15]. MEWS offers a proactive, evidence-based approach that can strengthen clinical decision-making, standardize monitoring, and facilitate timely intervention. Yet, its effectiveness in the Ethiopian context has not been assessed. This study, therefore, seeks to evaluate the impact of MEWS on predicting and reducing severe maternal outcomes in Ethiopian hospitals, generating context-specific evidence to inform, scale-up, and integrate into national maternal health strategies.

## Materials and methods

### Study design, setting, and population

A parallel, quasi-experimental study design was implemented in North Shewa Zone, Amhara Region, Ethiopia. The recruitment period was from 05/05/2025–31/8/2025. The Zone is bordered by the Oromia Region to the south and west, South Wollo to the north, and the Afar Region to the east. Debre Berhan is the capital city of the North Shewa Zone and is located about 130 kilometers northeast of Addis Ababa on the Ethiopian highway. The total population of the zone is estimated at 2,429,108, of which 1,203,366 are females [16]. There are eleven public hospitals in the zone: 3 General hospitals, 7 primary hospitals, and 1 comprehensive Specialized hospital.

### Population and eligibility criteria

The study included pregnant and postpartum women admitted with antepartum, intrapartum, or postpartum complications, as well as those with abortion-related cases, who had undergone obstetric or gynecologic surgery, and high-risk conditions. Women in normal labor who were planned for discharge within 24 hours after birth, those who died from accidental causes, and those transferred directly to the intensive care unit (ICU) without inpatient admission were excluded.

### Intervention

The intervention group was monitored using a statistically developed and validated Maternal Early Warning System (MEWS) chart (S1 File), which replaced the standard vital signs sheet for all enrolled participants at the intervention sites. The MEWS chart, previously validated and described in detail elsewhere [10,17], is a simple, observation-based tool designed to facilitate the early detection of maternal clinical deterioration. It categorizes parameters into three color-coded zones: Green (normal; no concern), Yellow (moderate abnormality; requires closer observation), and Red (severe derangement; demands immediate attention).

The chart incorporates twelve key maternal clinical parameters (Table 1). Monitoring follows an escalation protocol. If all parameters remained within the normal range (green), routine monitoring continued as per standard practice. A single yellow alert prompted repeat observations within 30 minutes while maintaining standard monitoring. A patient was considered triggered for further assessment if they received one red score or two yellow scores, in which case repeat observations were carried out within 30 minutes, followed by confirmation of findings through history and examination. Monitoring frequency was increased, and corrective measures such as administration of intravenous fluids, oxygen at 10 L/min if required, antihypertensives, review of charts, or appropriate maternal positioning (e.g., left tilt for pregnant women) were initiated. If the patient’s condition stabilized, routine monitoring was resumed; however, persistent or worsening red alerts required immediate review by a senior obstetrician within 60 minutes, which could lead to emergency intervention, urgent referral, or transfer to the intensive care unit (ICU). In cases where three or more yellow alerts or at least two red alerts were identified, immediate obstetrician review was mandatory, reassessment was conducted within 15 minutes, and continuous monitoring was started. If the situation remained unresolved, escalation of care to an anesthesiologist, critical care, and pain medicine specialist was required. This structured response system was designed to ensure timely recognition and rapid escalation of care, ultimately aiming to prevent progression to severe maternal morbidity or mortality (S3 File).

**Table 1:** Components of maternal early warning scores (MEWS) criteria for prediction of Severe Maternal Morbidity, Ethiopia, 2025.

Physiologic parameters	Normal values	Yellow alert	Red alert
Systolic blood pressure	100–150 mmHg	90–100 OR 150–160	<90 OR ≥160
Diastolic blood pressure	50–89 mmHg	90–110	≥110
Temperature	36–37.9 ◦C	35–36 ◦C	≤35 ◦C OR ≥38
Heart rate	50–99 BMP	40–50 OR 100–130	<40 OR>130
Respiratory rate	10–20 BMP	21–30	<10 OR>30
Oxygen saturation	95–100%	90–95%	<90%
Urine outputs	≥30 ml per hour	–	<35 ml/hour for 2 hours for catheterized women
Consciousness level	Alert	Responds to voice	To pain or unresponsive
Pain score	None to mild pain [0–3 pain score]	Intermittent pain at rest, moderate pain with movement [4–7 pain score]	Persistent pain at rest, severe pain with movement [8–10 pain score]
**For Postpartum or Post CS Women**			
Vaginal bleeding	No bleeding	Mild to moderate, i.e., spotting/light bleeding	Active, i.e., soaking a pad in <1 hour
Uterine contraction	Well-contracted uterus	Soft but responsive to massage	Boggy, poorly/ non-contracted uterus
Perineal tear	No tear	1^st^ and 2^nd^ degree tear	3^rd^ and 4^th^ degree tear

### Control group

The control hospitals were continuing with their existing/standard clinical monitoring practices. This involved monitoring and recording temperature, pulse, blood pressure, and respiratory rate on vital sign sheets.

### Clinical outcome

The primary clinical outcome was the number of patients identified by MEWS as at risk for severe maternal outcomes and the number who subsequently developed such outcomes. Secondary outcomes included intensive care unit (ICU) admissions, emergency surgical interventions, length of hospital stay, time to diagnose severe maternal morbidity (SMM), time to intervention, frequency of MEWS recording, and adherence to maternal monitoring through the use of the MEWS chart.

### Operational definition

**Triggering on MEWS chart:** A trigger is defined as a single markedly abnormal observation (red trigger) or a combination of two simultaneous mildly abnormal observations (two yellow triggers) [8].

**Severe maternal morbidity (SMM):** is a clinical condition or disease that can threaten a woman’s life during pregnancy and labor and after termination of pregnancy [18]. It was assessed using the Centers for Disease Control and Prevention (CDC) International Disease Classification indicators [19,20]. It includes hemorrhage, sepsis, pre-eclampsia, eclampsia, shock, acute renal failure, cardiovascular disorder (stroke), heart failure, severe anemia, pulmonary edema, hysterectomy, and thrombotic embolism.

**Maternal death:** death of a woman while pregnant or within 42 days of termination of pregnancy, irrespective of the duration and the site of the pregnancy, from any cause related to or aggravated by the pregnancy or its management, but not from accidental or incidental causes [18].

**Severe maternal outcome:** Severe maternal morbidity and maternal death [18].

**Composite maternal morbidity:** refers to the occurrence of one or more severe complications during pregnancy, childbirth, or within 42 days postpartum, including secondary outcomes [20].

**Emergency surgical interventions:** include emergency cesarean section, surgical repair of uterine rupture, hysterectomy, laparotomy, uterine artery ligation**,** compression sutures (e.g., b-lynch sutures), repair of perineal or vaginal tears, drainage of pelvic abscess or puerperal sepsis, and Wound Re-exploration for surgical site infection (SSI).

### Sample size

There was no comparable baseline study in Ethiopia. However, the incidence of severe maternal morbidity (SMM) in the study setting was 14.3% [21]. We hypothesize that the MEWS intervention would increase patients triggered for potential SMM by 21% [12]. To detect this difference at a 0.05 significance level with 80% power, and allowing for a 5% loss to follow-up, the study required a total of 1138 samples, 569 in the intervention and 569 in the control group.

### Study participants’ selection procedure

The study was conducted in four purposively selected hospitals. Debre Berhan University Hakim Gizaw Hospital and Debre Berhan Comprehensive Specialized Hospital (CSH) were designated as intervention sites, while Enat Hospital and Mehalmeda Hospital served as control sites. These hospitals were chosen because they are comparable in terms of service provision, the presence of obstetricians, availability of intensive care units, and their status as government facilities. The calculated sample was proportionally allocated to each hospital based on the six-month caseload of high-risk and postnatal care admissions. Study participants were then selected using a systematic random sampling method with a sampling interval of two, after the first participant was chosen by lottery in each hospital. Recruitment continued until the required sample size was achieved, including only women who met the eligibility criteria and consented to participate. Geographical separation between the hospitals helped minimize the risk of information contamination. At each hospital, all potentially eligible women were approached and informed about the study and care procedures before enrollment.

### Data collection tool and procedure

Midwives, interns, and general practitioners (GPs) were trained on the importance of accurately charting patient parameters and the mandatory requirement to call and involve an obstetrician whenever a trigger occurred. Compliance and completeness of MEWS documentation were regularly audited. To minimize observer bias, however, these staff members were not informed about the specific study objectives. Women were followed from enrollment until hospital discharge. In both intervention and control hospitals, monitoring of vital parameters was performed every six hours, or more frequently if indicated by the managing clinician. The frequency of observations was determined by the woman’s risk status, diagnosis, reason for admission, and initial assessment at admission. Individualized care plans specifying observation schedules were decided by the attending obstetrician. After childbirth or cesarean delivery, midwives used the MEWS chart to monitor women for two hours in the delivery room or post-anesthesia care unit (PACU) and continued monitoring in the postnatal or post-cesarean ward as per physician order or protocol.

The data collection tool was developed based on instruments applied in previous studies [6,10,17,20,22] (S2 File). Midwives conducted all MEWS assessments during inpatient care, identifying complications, detecting triggers, and ensuring timely clinical responses, with complete documentation in the medical record. Maternal outcome data and MEWS-related information were obtained through medical record reviews and face-to-face interviews. An independent data collector, blinded to group allocation, extracted outcome data from medical records within 24 hours of discharge.

### Data quality control

The questionnaire was translated from English to Amharic and back-translated to ensure consistency and preservation of meaning. A pretest involving 5% of the sample size (28 women from the intervention group and 28 from the control group) was conducted in a comparable facility to assess the validity and reliability of the tool, and necessary modifications were made based on the findings. Twelve BSc midwives (data collectors) and six MSc midwives (supervisors) were trained on the proper use of the MEWS chart, standardized clinical definitions, the data collection tool, and ethical considerations. Recruitment was conducted using neutral framing, and maternal outcome data were kept blinded from healthcare providers to reduce the Hawthorne effect. Both data collectors and providers were unaware of intervention and group assignments, and data collectors further minimized bias by building strong rapport with participants and spending adequate time with them.

During data collection, supervisors and investigators provided continuous on-site supervision, closely monitoring the completeness, accuracy, and clarity of the collected data. All parameters were measured using calibrated and validated equipment following standardized protocols to ensure accuracy and consistency. Data verification was performed through cross-checking with patient charts and periodic inter-observer validation. Monthly meetings were held to review adherence, address challenges, and implement corrective measures. In addition, women were anonymously surveyed to confirm adherence to key components, including pain management, vaginal bleeding, uterine contractions, and perineal tear monitoring.

### Data processing and analysis plan

The data were collected using the Kobo toolbox, exported to SPSS version 26, and cleaned for completeness, consistency, including identification and correction of missing and outlier values before analysis. Categorical variables were summarized using frequencies and percentages, while continuous variables were summarized using means with standard deviations and median with interquartile ranges. Proportions were compared using Pearson’s Chi-Square (χ2) test for categorical variables and the independent t-test for continuous variables. A generalized estimating equation (GEE) model was employed to investigate the difference in changes between the intervention and control groups. The effect of the MEWS intervention on binary outcomes, e.g., ICU admission, triggered cases, and SMM triggers, was estimated using the modified Poisson regression with a log link and robust (sandwich) standard errors, controlling for maternal and facility-level covariates (S1 Data). Results were reported as adjusted risk ratios with 95% confidence intervals, with a two-sided significance level of 0.05 (S1 Checklist).

### Ethical approval

The project was reviewed and ethical clearance was obtained from the Institutional Review Board of Debre Berhan University (Protocol number 01/2025 and Assigned number 19). The study was carried out following the guidelines of the Helsinki Declaration and adhering to the principles of Good Clinical Practice (GCP) [23]. A formal support letter was written to the study hospitals, and a permission letter was obtained from the hospital administrations. The data collectors explained the objective, potential risks, and benefits of the study to the study participants. Participants were informed that their participation was voluntary and that they could also withdraw from participating in the study at any time. Informed written consent was obtained from each participant. Those who cannot read and write were asked to thumbprint the consent form after the information was read. For participants younger than 18 years, written informed consent was obtained from their parents, and verbal assent was obtained from the minors themselves. Confidentiality and anonymity were assured, and the information was used only for research purposes.

### Trial registration

The trial was not registered prior to participant enrolment because the research team encountered delays during submission and the registration process, primarily due to incomplete information in the initial submission and the need to revise the application based on feedback from the registry. This back-and-forth communication and the need to begin the study on schedule prolonged approval beyond the planned study timeline, resulting in recruitment starting before formal registration was finalized. Despite this delay, the study adhered to all ethical requirements, including obtaining institutional approval and informed consent from participants. The authors confirm that all ongoing and related trials for this intervention are registered.

## Result

**Fig 1:**
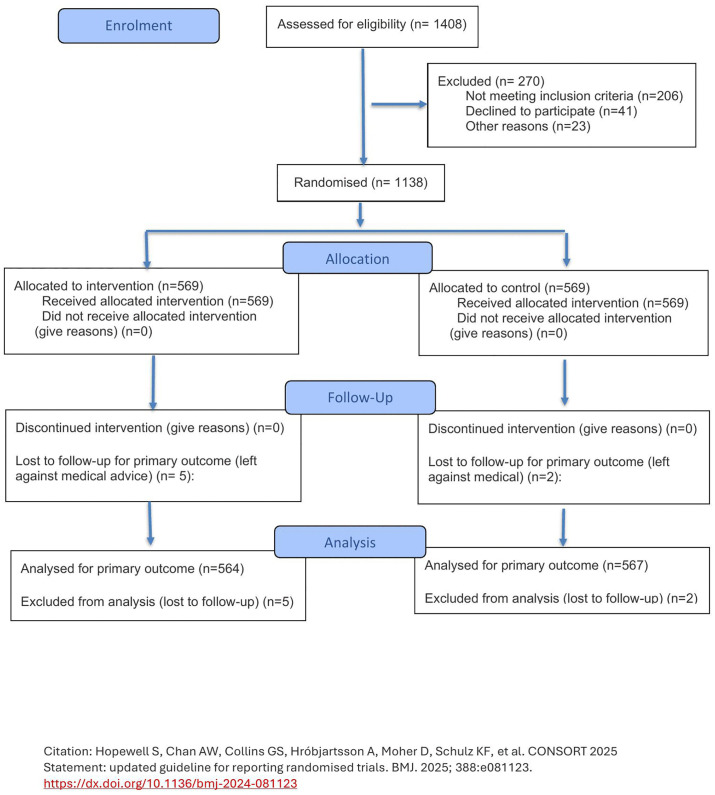
CONSORT flowchart of study participants, North Shewa Zone, Ethiopia, 2025.

During the recruitment period, 1408 pregnant women attended antenatal care clinics at the selected hospitals, of whom 1,202 were found eligible for the study. Among these, 127 opted out of participation, and 62 preferred to receive the standard care. Out of the 1138 women who met the inclusion criteria, 569 were allocated to the intervention group and 569 to the control group. In the intervention group, 5 participants were lost to follow-up, while 2 participants were lost to follow-up in the control group. Consequently, the final analysis included 564 participants in the intervention group and 567 in the control group (Fig 1).

### Socio-demographic characteristics of participants

No significant age difference was observed between the two groups. The mean age of participants in the control group was 27.7 ± 5.51 years, while in the intervention group, it was 28.5 ± 6.34 years. The majority of participants in both groups were in the 21–34-year age range. The number of women living in urban areas was higher in the control group (75.1%) compared to 63.8% in the intervention group. Additionally, there was a higher number of unemployed women in the control group (90.5%) compared to the intervention group (86.5%) (Table 2).

**Table 2:** Baseline characteristics of participants, North Shewa Zone, Amhara region, Ethiopia, 2025.

Variables	Category	Intervention	Control	p-value
		(N = 564)	(N = 567)	
**Age**	≤20 years	36 (6.4%)	63 (11.1%)	0.100
	21–34 years	442 (78.4%)	435 (76.7%)	
	≥35 years	86 (15.2%)	69 (12.2%)	
**Residence**	Rural	204 (36.2%)	141 (24.9%)	0.000
	Urban	360 (63.8%)	426 (75.1%)	
**Marital status**	Married	520 (92.2%)	543 (95.8%)	0.012
	Others*	44 (7.8%)	24 (4.2%)	
**Educational level**	No formal education	52 (9.2%)	96 (16.9%)	0.000
	Primary education	302 (53.5%)	255 (45.0%)	
	Secondary education	88 (15.6%)	135 (23.8%)	
	College and above	122 (21.6%)	81 (14.3%)	
**Occupation**	Employed	76 (13.5%)	54 (9.5%)	0.037
	Unemployed	488 (86.5%)	513 (90.5%)	
**Mode of transport to hospital**	Ambulance	46 (8.2%)	36 (6.3%)	0.000
	On foot	80 (14.2%)	45 (7.9%)	
	Private car	12 (2.1%)	0 (0.0%)	
	Public transport/Taxi	426 (75.5%)	486 (85.7%)	
**Health insurance (CBHI)**	No	450 (79.8%)	381 (67.2%)	0.000
	Yes	114 (20.2%)	186 (32.8%)	
**Distance to the hospital**	≤15 min	36 (6.4%)	69 (12.2%)	0.000
	16–30 min	258 (45.7%)	321 (56.6%)	
	>30 min	270 (47.9%)	177 (31.2%)	
**Referred from other facility**	No	392 (69.5%)	351 (61.9%)	0.007
	Yes	172 (30.5%)	216 (38.1%)	

*Widowed, Divorced, and Single

### Obstetric and reproductive characteristics

Women in the control group had a higher parity of 1–4 (82.0% compared to 73.8%), while a higher proportion of women in the intervention group reported a history of obstetric complications (14.2% compared to 8.5% in the control group). Twin pregnancies were more common in the control group (4.8% vs. 2.5%). Over half of the participants in both groups were admitted to the postnatal/post-CS ward (57.8% and 55.6%), and pre-existing medical illnesses were less frequent in the intervention group (2.8% compared to 8.5% in the control group) (Table 3).

**Table 3:** Obstetric and reproductive characteristics of participants, North Shewa Zone, Amhara region, Ethiopia, 2025.

Variables	Category	Intervention	Control	p-value
**N (%)**	**N (%)**
**Parity**	0	130 (23.0%)	84 (14.8%)	0.002
	1–4	416 (73.8%)	465 (82.0%)	
	≥5	18 (3.2%)	18 (3.2%)	
**History of obstetric complication**	No	484 (85.8%)	519 (91.5%)	0.002
	Yes	80 (14.2%)	48 (8.5%)	
**History of hospital admission**	No	514 (91.1%)	519 (91.5%)	0.811
	Yes	50 (8.9%)	48 (8.5%)	
**History of perinatal complication**	No	502 (89.0%)	501 (88.4%)	0.731
	Yes	62 (11.0%)	66 (11.6%)	
**Pregnancy type**	Singleton	550 (97.5%)	540 (95.2%)	0.04
	Twin	14 (2.5%)	27 (4.8%)	
**Ward location of participants**	Gynecology	174 (30.9%)	48 (8.5%)	0.000
	High-risk	52 (9.2%)	195 (34.4%)	
	Labor	12 (2.1%)	9 (1.6%)	
	Postnatal/Post-CS	326 (57.8%)	315 (55.6%)	
**Pre-existing medical illness**	No	548 (97.2%)	519 (91.5%)	0.000
	Yes	16 (2.8%)	48 (8.5%)	
**Alcohol or another drug use**	No	560 (99.3%)	495 (87.3%)	0.000*
	Yes	4 (0.7%)	72 (12.7%)	
**Delay in hospital care**	No	564 (100%)	504 (88.9%)	0.000*
	Yes	0 (0.0%)	63 (11.4%)	

*Fisher’s Exact Test CS: Cesarean section

### MEWS triggering and monitoring and maternal outcomes

Complete chart documentation was considerably higher in the intervention group (97.5%) than in the control group (31.2%). Triggered cases were more frequent among the intervention group (11.7%) compared to controls (7.9%). Additionally, 57.6% of women in the intervention group had two or more red score alerts versus 40.0% in the control group. The most common alert finding in the MEWS group was a combination of pulse rate, uterine contraction, and vaginal bleeding (15.2%), while in the control group, it was systolic and diastolic blood pressure (86.6%). Severe maternal outcomes (SMOs) occurred in 1.1% of women in the intervention group compared to 5.3% in controls. Monitoring frequency also differed significantly, with most patients in the intervention group monitored every 15–30 minutes (78%), whereas 41.3% of women in the control group were monitored only once per shift (Table 4).

**Table 4:** MEWS triggering and monitoring of participants, North Shewa Zone, Amhara region, Ethiopia, 2025.

Variables	Category	Intervention	Control	p-value
		N (%)	N (%)	
**Complete chart filled**	No	14 (2.5%)	390 (68.8%)	0.000
	Yes	550 (97.5%)	177 (31.2%)	
**Triggered patients**	No	498 (88.3%)	522 (92.1%)	0.033
	Yes	66 (11.7%)	45 (7.9%)	
**Alert scores for triggered patients**	≥2 red scores	38 (57.6%)	18 (40.0%)	0.008
**(N=111)**	≥2 yellow scores	8 (12.1%)	15 (33.3%)	
	1 red scores	20 (30.3%)	12 (26.7%)	
**Alert findings (N=111)**	PR and SPO2	2 (3.0%)	0 (0.0%)	0.000
	PR and T◦	6 (9.1%)	0 (0.0%)	
	PR and VB	4 (6.1%)	0 (0.0%)	
	PR, SBP, and VB	2 (3.0%)	0 (0.0%)	
	PR, Ux contr. and VB	10 (15.2%)	0 (0.0%)	
	PR, VB, and T◦	2 (3.0%)	0 (0.0%)	
	SBP	10 (15.2%)	3 (6.7%)	
	SBP and DBP	12 (18.2%)	39 (86.6%)	
	SBP and RR	2 (3.0%)	0 (0.0%)	
	SBP, DBP, and PR	2 (3.0%)	0 (0.0%)	
	SBP, DBP, T◦ and Pain	0 (0.0%)	3 (6.7%)	
	SBP, PR, VB, and Ux contr.	2 (3.0%)	0 (0.0%)	
	SPO2	2 (3.0%)	0 (0.0%)	
	T◦	6 (9.1%)	0 (0.0%)	
	VB	2 (3.0%)	0 (0.0%)	
	VB, T◦, SBP, DBP, and SPO2	2 (3.0%)	0 (0.0%)	
**Physician consultation**	No	0 (0.0%)	3 (6.7%)	0.012*
	Yes	66 (100.0%)	42 (93.3%)	
**Actions taken following triggers**	Yes	66 (100.0%)	45 (100.0%)	0.036*
	No	0 (0.0%)	0 (0.0%)	
**Escalation protocol followed appropriately**	No	2 (3.0%)	3 (6.7%)	0.070*
	Yes	64 (97.0%)	42 (93.3%)	
**Severe maternal outcomes (SMO)**	No	558 (98.9%)	537 (94.7%)	0.000
	Yes	6 (1.1%)	30 (5.3%)	
**Identified SMOs**	Anemia	0 (0.0%)	18 (60.0%)	0.000
	Eclampsia	2 (33.3%)	0 (0.0%)	
	Hemorrhage	0 (0.0%)	3 (10.0%)	
	Postpartum hemorrhage	2 (33.3%)	0 (0.0%)	
	Sepsis	0 (0.0%)	3 (10.0%)	
	Severe pre-eclampsia	0 (0.0%)	3 (10.0%)	
	Uterine rupture	1 (16.7%)	3 (10.0%)	
	Maternal death	1 (16.7%)	0 (0.0%)	
**Patient readmission**	No	556 (98.6%)	552 (97.4%)	0.144
	Yes	8 (1.4%)	15 (2.6%)	
**ICU admission**	No	562 (99.6%)	561 (98.9%)	0.158*
	Yes	2 (0.4%)	6 (1.1%)	
**Frequency of patient monitoring**	Every 15 minutes	46 (8.2%)	0 (0.0%)	0.000*
	Every 30 minutes	440 (78.0%)	162 (28.6%)	
	Every 4 hours	60 (10.6%)	132 (23.3%)	
	Every 6 hours	18 (3.2%)	39 (6.9%)	
	Once per shift	0 (0.0%)	234 (41.3%)	

*Fisher’s Exact Test PR: Pulse rate T◦: Temperature VB: Vaginal bleeding RR: Respiratory rate Ux contr.: Uterine contraction SBP: Systolic blood pressure DBP: Diastolic blood pressure SPO2: Oxygen saturation

### Association of MEWS intervention with clinical monitoring

The independent samples t-test was performed to compare key clinical monitoring indicators between women monitored using the MEWS chart and those receiving standard care. The mean duration from admission to the first trigger was shorter in the intervention group (5.61 ± 2.81 hours) compared to the control group (10.27 ± 3.49 hours, *p* < 0.001). Similarly, the mean time from trigger to physician evaluation was lower in the MEWS group (49.30 ± 13.28 minutes) than those receiving standard care (71.87 ± 14.78 minutes *p* < 0.001). The mean time from trigger to clinical intervention was also shorter in the intervention group (14.58 ± 7.86 minutes) compared to the control group (25.87 ± 9.23 minutes, *p* < 0.001). Women in the control group underwent a higher mean number of ultrasound scans (2.30 ± 1.92) than those in the intervention group (1.32 ± 1.46, *p* < 0.001)). Additionally, the mean length of hospital stay was shorter among patients in the intervention group (4.83 ± 7.51 days) compared to those in the control group (5.29 ± 8.80 days, *p* < 0.001)). Although women in the intervention group had shorter mean durations from admission to severe maternal outcome (8.00 ± 3.89 vs. 13.70 ± 14.19 hours) and from trigger to diagnosis of severe maternal outcome (2.33 ± 1.03 vs. 7.00 ± 8.02 hours) compared with the control group, these differences were not statistically significant (*p* = 0.341 and *p* = 0.169, respectively) (Table 5).

**Table 5:** The effect of MEWS triggering and monitoring, North Shewa Zone, Amhara Region, Ethiopia, 2025.

Variables	Category	Intervention N (%)	Control N (%)	p-value
**Hours from admission to first trigger**	Mean ± SD	5.61 ± 2.81	10.27 ± 3.49	0.000
**Minutes from trigger to physician evaluation**	Mean ± SD	49.30 ± 13.28	71.87 ± 14.78	0.000
**Minutes from trigger to clinical intervention**	Mean ± SD	14.58 ± 7.86	25.87 ± 9.23	0.000
**Hours from admission to SMO**	Mean ± SD	8.00 ± 3.89	13.7 ± 14.19	0.341
**Hours from trigger to diagnosis of SMO**	Mean ± SD	2.33 ± 1.03	7.00 ± 8.02	0.169
**Number of ultrasound scans**	Mean ± SD	1.32 ± 1.46	2.30 ± 1.92	0.000
**Length of hospital stay in days**	Mean ± SD	4.83 ± 7.51	5.29 ± 8.80	0.000

SD: Standard deviation SMO: Severe maternal outcomes

### Association of MEWS monitoring with Maternal outcomes

After adjustment for potential confounders, women monitored using the MEWS chart were observed to have a 20% lower risk of severe maternal outcomes compared to those who received standard care (aRR = 0.85, 95% CI: 0.73–0.99). Furthermore, women in the MEWS-monitored group had a 9% higher likelihood of being triggered for timely clinical response than those in the control group (aRR = 1.15, 95% CI: 1.03–1.28) (Table 6).

**Table 6:** Effect of MEWS intervention on maternal outcomes in North Shewa Zone, Amhara region, Ethiopia, 2025.

Variables	Category	Intervention N (%)	Control N (%)	CRR (95% CI)	ARR (95% CI) ᵃ
**Severe maternal outcomes (SMO)**	Yes	6 (1.1%)	30 (5.3%)	0.77 (0.68-0.88)	0.85 (0.73-0.99) *
	No	558 (98.9%)	537 (94.7%)	1	1
**Triggered patients**	Yes	66 (11.7%)	45 (7.9%)	1.07 (1.02-1.12)	1.15 (1.03-1.28) *
	No	498 (88.3%)	522 (92.1%)	1	1
**Readmissions**	Yes	8 (1.4%)	15 (2.6%)	0.89 (0.89-0.90)	0.97 (0.93-1.02)
	No	556 (98.6%)	552 (97.4%)	1	1

ᵃAdjusted for age, residence, marital status, educational level, occupation, community-based health insurance, distance to hospital, being referred from another facility, income, parity, history of obstetric complication, history of hospital admission, history of fetal/ perinatal complication, pregnancy type, ward location, alcohol/ other drug use and pre-existing medical illness.

*P value <0.005

## Discussion

This study aimed to evaluate the MEWS model in predicting and reducing severe maternal outcomes. The duration from admission to the first trigger was reduced by 4.7 hours, time to physician evaluation by 22.6 minutes, and time to clinical intervention by 11.3 minutes in the intervention group. Women also had on average one fewer ultrasound scan per patient and a shorter hospital stay by about 0.5 days. Women monitored with the MEWS chart had a 20% lower adjusted risk of developing severe maternal outcomes. Furthermore, MEWS-monitored women were 9% more likely to be triggered for timely clinical response.

In this study, the duration from admission to the first trigger was reduced by approximately 4.7 hours in the MEWS group. The system is associated with earlier identification of maternal deterioration compared to standard care. By systematically monitoring physiologic parameters and assigning scores to deviations, MEWS may facilitate timely recognition of risk, allowing healthcare providers to intervene before complications escalate. Supporting this, evidence from India shows that MEWS enabled early recognition of obstetric morbidity even before clinical signs and symptoms became evident [24]. Together, these findings underscore that MEWS is a useful tool associated with earlier detection, prompt intervention, and reduced severe maternal outcomes, enhancing overall maternal safety and quality of care [22].

The reduction in the time from trigger to physician evaluation by approximately 22.6 minutes (49.30 vs. 71.87 minutes) indicates that implementation of the MEWS system is associated with more timely clinical response once early warning criteria are met. This shorter interval suggests that abnormal maternal conditions are recognized and escalated to physicians more rapidly, allowing for prompt assessment and intervention, which is crucial in preventing progression to severe maternal outcomes. Similar findings have been reported in other studies. For instance, a systematic review highlighted that MEWS increases the frequency of monitoring and facilitate timely clinical actions for abnormal observations, thereby reducing delays in physician assessment [9]. Another review found that MEWS reduces the time between detection of abnormal clinical parameters and the initiation of corrective interventions, emphasizing its role in enhancing patient safety and reducing maternal morbidity [13]. These findings align with global evidence indicating that structured MEWS support efficient communication among healthcare teams and accelerate the delivery of critical care, particularly in obstetric settings where rapid deterioration can occur.

The time from trigger to clinical intervention was reduced by nearly 11.3 minutes (14.58 vs. 25.87 minutes). This demonstrates that the MEWS system is associated with faster clinical response once abnormal vital signs are detected. By providing a structured scoring system and clearly defined escalation thresholds, MEWS ensures that abnormal observations prompt immediate action, minimizing delays in management. This is supported by systematic reviews showing that MEWS increases the frequency of monitoring and facilitates prompt clinical action for abnormal observations [9], and that it reduces the interval between detection of abnormal parameters and corrective interventions [13]. The findings highlight the association between MEWS use and improved response times and enhanced patient safety in obstetric care.

Additionally, women in the intervention group underwent fewer ultrasound scans, with a reduction of approximately one scan per patient. This finding suggests that the use of MEWS for early detection and timely clinical management may be associated with reduced need for additional diagnostic investigations. By identifying maternal deterioration promptly and facilitating immediate interventions, clinicians may have been able to address complications before they escalated, thereby limiting reliance on repeated imaging to assess maternal or fetal status. This not only reduces patient exposure to unnecessary procedures but may also contribute to more efficient use of healthcare resources.

Women who were followed using the MEWS chart had a shorter mean hospital stay by approximately 0.5 days. This suggests that early detection of clinical deterioration and timely intervention may be associated with shorter hospital stay, possibly by preventing complications from progressing and allowing more efficient management of maternal conditions. By identifying abnormal signs early, healthcare providers can implement appropriate treatment promptly, which may reduce the overall duration of hospitalization. Previous studies have similarly shown that MEWS is associated with improved patient outcomes by enabling timely clinical response, which can contribute to shorter hospital stays and more effective use of meager healthcare resources.

Women monitored with the MEWS chart had a lower adjusted risk of developing severe maternal outcomes compared to those receiving standard care. Similarly, in the USA, use of the MEWS tool resulted in significant reductions in CDC severe maternal morbidity and composite morbidity [20]. These data support the recommendations from the Alliance For Innovation On Maternal Health (AIM) that the tool should be used to improve timely assessment and treatment of maternity patients [25]. They are also consistent with others who have reported that maternal early warning tools are associated with reduced maternal morbidity [7,8,26]. The MEWS tool enables early detection of clinical deterioration through systematic and objective assessment of parameters. MEWS facilitates the timely recognition of abnormal physiological parameters. By triggering an early clinical response and escalation of care, it allows healthcare providers to intervene before complications progress to life-threatening stages.

Furthermore, women in the MEWS group were more likely to be triggered for timely clinical assessment and intervention. The MEWS chart provides a standardized, objective scoring system that identifies deviations from normal vital signs promptly. This ensures that even subtle signs of deterioration are recognized early, prompting immediate clinical review and intervention. As a result, healthcare providers can act proactively rather than reactively, preventing progression to severe maternal complications. This finding is consistent with evidence from Spain, where the implementation of MEWS in the early postpartum period resulted in a higher number of triggered patients, demonstrating that structured early warning systems enhance detection and response to maternal instability [10]. By facilitating systematic monitoring, MEWS improves patient safety, timely decision-making, and overall maternal outcomes compared to standard care.

### Clinical implication

The implementation of the MEWS chart in obstetric care has several important clinical implications. First, it facilitates early detection of maternal deterioration, as evidenced by the reduced time from admission to the first trigger and from trigger to physician evaluation. This allows healthcare providers to intervene promptly, which may help reduce progression to severe maternal outcomes. Second, the MEWS system improves the timeliness and efficiency of clinical response, as shown by shorter intervals to clinical intervention and physician assessment. This ensures that abnormal physiological changes are addressed quickly, which may be associated with reduced morbidity and potentially prevent life-threatening complications.

Third, early recognition and intervention appear to reduce the need for additional investigations, such as ultrasound scans, suggesting more targeted and efficient clinical management. Similarly, the shorter hospital stays in the intervention group indicate that prompt management may be associated with faster recovery, improve patient flow, and optimize resource utilization in healthcare settings. Finally, the findings suggest that MEWS can enhance overall patient safety, clinical decision-making, and quality of care in obstetric settings. Incorporating MEWS into routine maternal monitoring may help standardize assessments, improve communication among healthcare teams, ensure timely escalation of care, and may be associated with a lower risk of severe maternal complications, particularly in resource-limited or high-volume hospitals.

### Strengths and limitations

The strength of our study lies in its prospective design and the inclusion of a relatively large study population. A key limitation of this study is the small number of clusters (four hospitals), which may have contributed to baseline imbalances in participant characteristics between study groups and limited the precision of variance estimates obtained from GEE analyses. Although GEE was used to account for within-hospital correlation, it does not eliminate potential confounding associated with cluster-level treatment assignment. Therefore, residual confounding cannot be completely excluded, and the observed differences between groups should be interpreted as associational rather than causal. Another, limitation is the low number of women with specific morbidity outcomes, which prevented separate analyses for each type of morbidity. Additionally, there is no universally accepted early warning system for the obstetric population, and physiological and threshold values may vary across settings. We also did not differentiate between high-risk women (those with morbidity) and low-risk women (those without morbidity) when applying the MEWS chart. As a result, the observed mean differences in physiological parameters between the intervention and control groups may be underestimated relative to what might be expected in a stratified or risk-adjusted context. Further, trial registration was completed after participant recruitment had commenced.

## Conclusion and recommendation

The use of the MEWS chart in obstetric care was associated with earlier detection of maternal deterioration, faster clinical response, fewer unnecessary investigations, improved communication among teams, and shorter hospital stays. These results indicate that MEWS may be associated with improved maternal outcomes and enhance the efficiency of care. It is recommended that MEWS be considered for integration into the routine monitoring of hospitalized women to facilitate the timely identification and management of complications. Further research involving a larger number of clusters should explore its application in different risk groups and settings to optimize its effectiveness.

## Supporting information

S1 File MEWS follow-up chart. (PDF)

S2 File English version questionnaire. (PDF)

S3 File Study protocol used for the present study. (DOCX)

S1 Checklist TREND statement checklist. (PDF)

S1 Data Raw dataset used in the present study. (SAV)

## Abbreviations

DBU: Debre Berhan University
EDHS: Ethiopian Demographic and Health Survey
IPHC-E: International Institute for Primary Health Care-Ethiopia
MEWS: Maternal Early Warning System
VS: Vital sign
