# Supplementary material for: Maternal early warning system (MEWS) model for predicting and reducing severe maternal outcomes in Ethiopia
Source: PLoS One. 2026 Aug 14;21(8):e0356105. doi: 10.1371/journal.pone.0356105 (PMC13475879; doi:10.1371/journal.pone.0356105)
Supplement: Supplementary file 1 [file pone.0356105.s001.pdf]

Maternal Early Warning Score (MEWS) Chart for Clinical Monitoring and Intervention

Patient name: ________________________________

Date of admission: ___________ **Time:** __________

Ward: ______________________________________

Diagnosis: ___________________________________

Medical record number (MRN): _______________

- **If all parameters remain normal**: routine monitoring continues as per standard practice or the physician's order.
- **If 1 yellow alert:** repeat observations in 30 minutes and continue monitoring.
- **If 2 yellow alerts or 1 red alert:** repeat observations in 30 minutes and if persists or worsens, consult the obstetrician.
- **If ≥ 3 yellow alerts or ≥ 2 red alerts:** immediate review by obstetrician and repeat observations in 15 minutes or continuous monitoring.

Date	Time		Sys BP	Dys BP	Temp	Pulse	Resp	O_2 sat.	Urine out put	Cons. level	Pain scor	For PP/ Post-CS women			Trigger (Yes/ No)	Review time if trigger	Sign
												Vaginal bleeding	Ux. contr	P. tear			
		Obser.															
		Alert															
		Obser.															
		Alert															
		Obser.															
		Alert															
		Obser.															
		Alert															
		Obser.															
		Alert															
		Obser.															
		Alert															
		Obser.															
		Alert															

BP: Blood pressure Cons.: consciousness Obs.: Observation Ux contr: Uterine contraction P. tear: Perineal tear PP: Postpartum

Note: A patient is considered **triggered** for further assessment if they receive **one red score or two yellow scores**.

- **Alert:** Green, Yellow, or Red alert.
