# Supplementary material for: Maternal early warning system (MEWS) model for predicting and reducing severe maternal outcomes in Ethiopia
Source: PLoS One. 2026 Aug 14;21(8):e0356105. doi: 10.1371/journal.pone.0356105 (PMC13475879; doi:10.1371/journal.pone.0356105)
Supplement: Supplementary file 2 [file pone.0356105.s002.pdf]

English version questionnaire

Questionnaire code: ______________ Hospital: __________________ MRN: _______________

Part-I: Socio-demographic characteristics

S. No	Questions	Responses options	Skip
1.	What is your age	________Years	
2.	What is your place of residence?	1. Urban<br>2. Rural	
3.	What is your marital status?	1. Single<br>2. Married<br>3. Divorced<br>4. Widowed	
4.	What is your educational level?	1. No formal education<br>2. Primary education<br>3. Secondary education<br>4. College and above	
5.	What is your occupation?	1. Employed<br>2. Unemployed<br>3. Retired	
6.	How many minutes does it take to reach the hospital from your home?	__________ minutes.	
7.	What was type of transport did you use to reach the hospital?	1. Ambulance<br>2. Public transport/ Taxi/ Bajaj<br>3. Private car<br>4. On foot	
8.	Do you have community-based health insurance (CBHI)	1. Yes<br>2. No	
9.	How many children do you have?	_____________	
10.	What is your family size?	_____________	
11.	Were you referred from another health facility?	1. Yes<br>2. No	
12.	What is your average monthly household income?	______________ Birr.	

Part II: Obstetric and reproductive health factors

S. No	Questions	Responses options	Skip
1.	How many times have you been pregnant (Gravidity)?	__________	
2.	How many births have you had after 28 weeks including both alive and stillbirths (Parity)?	__________	
3.	Did you attend antenatal care (ANC) during this pregnancy?	1. Yes<br>2. No	If "No" skip to Q6
4.	If **Yes**, how many ANC visits did you attend during this pregnancy?	__________times	
5.	At what week did you start ANC in this pregnancy?	__________ weeks	
6.	Do you have history of Maternal obstetric complication in previous pregnancies?	1. Yes<br>2. No	If "No" skip to Q8
7.	If **Yes**, select all that apply (More than one answer is allowed)	1. Pre-eclampsia<br>2. PROM<br>3. Hemorrhage<br>4. Sepsis<br>5. Anemia<br>6. ICU admission<br>7. GDM<br>8. Oligohydramnios<br>9. Others____________	
8.	Do you have any previous history of hospital admission related to pregnancy or childbirth complications?	1. Yes<br>2. No	If "No" skip to Q10
9.	If yes, what was the specific complication or reason for the admission?	1. Pre-eclampsia<br>2. PROM<br>3. Antepartum hemorrhage<br>4. Sepsis<br>5. Anemia<br>6. Preterm labor<br>7. GDM<br>8. Oligohydramnios<br>9. Surgical site infection<br>10. Other specify __________	

10.	Do you have history of perinatal complication in previous pregnancy?	1. Yes<br>2. No	If "No" skip to Q12
11.	If **Yes**, select (More than one answer is allowed)	1. Abortion<br>2. Stillbirth<br>3. Neonatal death<br>4. Fetal growth restriction<br>5. Low birth weight<br>6. Macrosomia<br>7. Prematurity<br>8. Congenital anomaly<br>9. Perinatal asphyxia<br>10. Meconium aspiration syndrome<br>11. Admission to NICU<br>12. Need for resuscitation	
12.	Current pregnancy type	1. Singleton<br>2. Twin	
13.	Location of mother's ward	1. High risk (not in labor)<br>2. Labor<br>3. Postpartum/Post CS	
14.	Do you have any known pre-existing medical illness?	1. Yes<br>2. No	If "No" skip to Q16
15.	If **Yes,** please specify the disease? (More than one answer is allowed)	1. Hypertension<br>2. Diabetes<br>3. Cardiac disease<br>4. Kidney disease<br>5. Liver disease<br>6. HIV/AIDS<br>7. Others ____________	
16.	What was the interpregnancy interval (duration between the last date of birth and LNMP of this pregnancy) in months?	__________ months.	
17.	What was the diagnosis at admission?	1. Pre-eclampsia<br>2. PROM<br>3. Hemorrhage<br>4. Sepsis	

		5. Labor and delivery<br>6. Anemia<br>7. Gestational diabetes<br>8. Others specify__________	
18.	Gestational age at admission	_________ weeks.	
19.	Gestational age at delivery	__________ weeks	
20.	Was the current pregnancy planned?	1. Yes<br>2. No	
21.	Do you use alcohol or other drugs?	1. Yes<br>2. No	
22.	Was there any delay in receiving care at the facility?	1. Yes<br>2. No	
23.	Was there any experience of physical or emotional abuse during pregnancy?	1. Yes<br>2. No	

Part III: MEWS triggering and monitoring

S. No	Questions	Responses options	Skip
1.	Type of monitoring used:	1. MEWS chart<br>2. Standard clinical monitoring (Vital sign sheet)	If "2" skip to Q17
2.	Was the MEWS chart filled completely?	1. Yes<br>2. No	
3.	Did the patient have abnormal MEWS finding (≥1 red score or ≥2 yellow scores)?	1. Yes<br>2. No	If No skip to Q17
4.	If Yes, what was the alert finding for a triggered patient?	1. ≥2 yellow scores<br>2. 1 red score<br>3. ≥2 red scores	
5.	What was the alert finding (Sys BP, Dys BP, HR, O_2 sat…)	_________________	
6.	What was the duration from symptom onset to the MEWS trigger?	___________ hours	
7.	What was the duration from admission to the first MEWS trigger? (in hours)	___________ hours	
8.	What was the suspected complication based on the trigger?	1. Pre-eclampsia<br>2. Eclampsia	

		3. Hypovolemia/hypotension<br>4. PPH<br>5. Sepsis<br>6. Anemia<br>7. Magnesium sulfate toxicity<br>8. Pulmonary edema<br>9. Pulmonary embolism<br>10. Chorioamnionitis<br>11. Surgical site infection<br>12. Other specify__________	
9.	Was a physician consulted in response to the MEWS trigger?	1. Yes<br>2. No	If "No" skip to Q12
10.	If **Yes**, what was the physician's diagnosis after the consultation?	_________________	
11.	What was the time interval between the MEWS trigger and the physician's evaluation following consultation?	___________ minutes.	
12.	Was clinical action taken in response to the MEWS trigger?	1. Yes<br>2. No	If "No" skip to Q15
13.	What type of action was taken in response to MEWS triggering?	1. Continuous monitoring only<br>2. Repeat observations/ monitoring in 30 minutes.<br>3. Repeat observations/ monitoring in 15 minutes.<br>4. Blood transfusion<br>5. Resuscitation (O_2 administration, IV fluid, and positioning)<br>6. Medication prescription<br>7. Emergency surgical intervention<br>8. Transfer to ICU<br>9. Referral to another facility<br>10. No action taken<br>11. Other specify___________	

14.	If you select emergency surgical intervention, please specify?	1. Emergency cesarean section<br>2. Hysterectomy<br>3. Laparotomy<br>4. MVA (D and E/suction)<br>5. Surgical repair of uterine rupture<br>6. Uterine artery ligation<br>7. Compression sutures (e.g., b-lynch sutures)<br>8. Repair of perineal or vaginal tears<br>9. Drainage of pelvic abscess or puerperal sepsis.<br>10. Wound Re-exploration for SSI<br>11. Other (specify): _______	
15.	What was the duration from MEWS trigger (abnormal MEWS) to clinical intervention / action? (in minutes)	__________ minutes.	
16.	Was MEWS escalation protocol followed appropriately?	1. Yes<br>2. No	
17.	Was the vital sign sheet filled completely?	1. Yes<br>2. No	
18.	Were abnormal vital signs obtained?	1. Yes<br>2. No	If No skip to Q30
19.	What was the abnormal vital sign finding (Sys BP, Dys BP, HR, RR, T^o, O_2 sat…)	________________	
20.	What was the duration from symptom onset to the abnormal vital sign record?	__________ hours	
21.	What was the duration from admission to the abnormal vital sign? (in hours)	__________ hours	
22.	What was the suspected complication based on the abnormal vital sign finding?	1. Pre-eclampsia<br>2. Eclampsia<br>3. Hypovolemia/hypotension<br>4. Hemorrhage<br>5. Sepsis<br>6. Anemia	

		7. Magnesium sulfate toxicity<br>8. Pulmonary edema<br>9. Pulmonary embolism<br>10. Chorioamnionitis<br>11. Surgical site infection<br>12. Other specify__________	
23.	Was a physician consulted in response to the abnormal vital sign?	1. Yes<br>2. No	If "No" skip to Q26
24.	If **Yes**, what was the physician's diagnosis after the consultation?	_________________	
25.	What was the time interval between the abnormal vital sign and the physician's evaluation following consultation?	___________ minutes.	
26.	Was clinical action taken in response to the abnormal vital sign sheet trigger?	1. Yes<br>2. No	If "No" skip to 30
27.	What type of action was taken in response to abnormal vital sign trigger?	1. Continuous monitoring only<br>2. Repeat observations/ monitoring in 30 minutes.<br>3. Repeat observations/ monitoring in 15 minutes.<br>4. Blood transfusion<br>5. Resuscitation (O_2 administration, IV fluid, and positioning)<br>6. Medication prescription<br>7. Emergency surgical intervention<br>8. Transfer to ICU<br>9. Referral to another facility<br>10. No action taken<br>11. Other specify___________	
28.	If you select emergency surgical intervention, please specify?	1. Emergency cesarean section<br>2. Hysterectomy<br>3. Laparotomy<br>4. MVA (D and E/suction)	

		5. Surgical repair of uterine rupture<br>6. Uterine artery ligation<br>7. Compression sutures (e.g., b-lynch sutures)<br>8. Repair of perineal or vaginal tears<br>9. Drainage of pelvic abscess or puerperal sepsis.<br>10. Wound Re-exploration for SSI<br>11. Other (specify): ________	
29.	What was the duration from the abnormal vital sign trigger to clinical intervention / action? (in minutes)	___________ minutes.	
30.	Number of ultrasound scans after admission?	____________	

Part IV: Severe Maternal Morbidity and Mortality

S. No	Questions	Responses options	Skip
1.	Did the women develop any form of Severe Maternal Morbidity (SMM) during her hospital stay after admission to the ward?	1. Yes<br>2. No	If "No" skip to part V
2.	If **Yes**, please specify**?** *(More than one answer is allowed)*	1. Hemorrhage<br>2. Severe pre-eclampsia<br>3. Eclampsia<br>4. Sepsis<br>5. Anemia<br>6. Uterine rupture<br>7. Pulmonary edema<br>8. Respiratory distress<br>9. Need for blood transfusion<br>10. Admission to ICU<br>11. Hysterectomy<br>12. Organ failure<br>13. Maternal death<br>14. Other specify_______	

3.	When did the woman develop Severe Maternal Morbidity (SMM) after being admitted?	__________ hours.	
4.	What was the duration from MEWS trigger/ abnormal vital sign record to the diagnosis of SMM?	__________ hours.	
5.	Was the MEWS trigger/ abnormal vital sign record predictive of the SMM condition?	1. Yes<br>2. No	

Part V: Clinical Outcome Comparisons

S. No	Questions	Responses options	Skip
1.	Length of hospital stay (in days)	_________ days	
2.	Was the patient readmitted for maternal complications after discharge?	1. Yes<br>2. No	
3.	Was the patient admitted to the Intensive Care Unit (ICU)?	1. Yes<br>2. No	
4.	How frequently was the patient monitored either using the MEWS chart or vital sign sheet?	1. Every 4 hours<br>2. Every 6 hours<br>3. Once per shift<br>4. Irregularly<br>5. Not documented<br>6. Other specify____________	
7.	Outcome at discharge:	1. Stable, discharged home<br>2. Transferred to ICU<br>3. Referred to another facility<br>4. Maternal death<br>5. Discharged against medical advice<br>6. Lost to follow-up	

Thank you very much!!!
