# Supplementary material for: Maternal early warning system (MEWS) model for predicting and reducing severe maternal outcomes in Ethiopia
Source: PLoS One. 2026 Aug 14;21(8):e0356105. doi: 10.1371/journal.pone.0356105 (PMC13475879; doi:10.1371/journal.pone.0356105)
Supplement: Supplementary file 4 [file pone.0356105.s003.docx]

**Debre Berhan University, Asrat Woldeyes Health Science Campus**

**Maternal Early Warning System (MEWS) Model for predicting severe maternal morbidity and reducing maternal mortality in Ethiopia, 2025: Implementation research**

**Investigators:**

1. Saba Desta (MSc in Clinical Midwifery)
2. Mesfin Tadese (MSc in Clinical Midwifery)
3. Dr. Solomon Hailemeskel Beshah (PhD in Women’s Health)
4. Chaltu Takele (MPH in Reproductive Health)
5. Dr. Getaneh Dejen (Obstetrician and Gynecologist)
6. Dr. Lidya Asalefew (MD, General Practitioner)
7. Getnet Mitike Kassie (Senior Research advisor, International Institute for Primary Health Care – Ethiopia)

**Proposal submitted to Debre Berhan University, Asrat Woldeyes Health Science Campus, Research, Community Service, and CBE Director.**

**April 2025**

**Debre Berhan, Ethiopia**

# Investigators Affiliation

**Name, Departments, and Address of Investigators**

| **S.No** | **Name** | **Department** | **Email** | **Telephone** |
| --- | --- | --- | --- | --- |
| 1. | Saba Desta | AWHSC, Midwifery | [sabadesta127@gmail.com](mailto:sabadesta127@gmail.com) | 0911116065 |
| 2. | Mesfin Tadese | AWHSC, Midwifery | [mesitad031@gmail.com](mailto:mesitad031@gmail.com) | 0915839921 |
| 3. | Dr. Solomon Hailemeskel | AWHSC, Midwifery | [solomonhailemeskel9@gmail.com](mailto:solomonhailemeskel9@gmail.com) | 0913312912 |
| 4. | Chaltu Takele | AWHSC, Public Health | [chaltutakele266@gmail.com](mailto:chaltutakele266@gmail.com) | 0932456109 |
| 5. | Dr. Getaneh Dejen | AWHSC, Medicine | [legetaneh@dbu.edu.et](mailto:legetaneh@dbu.edu.et) | 0913292030 |
| 6. | Dr. Lidya Asalefew | AWHSC, Medicine | [lidoase@gmail.com](mailto:lidoase@gmail.com) | 0985396424 |
| 7. | Prof. Getnet Mitike kassie | IPHC-E | [getnet.mitike@iphce.org](mailto:getnet.mitike@iphce.org) | 0957043005 |

# Acknowledgment

We express our gratitude to IPHC-E, the International Institute for Primary Health Care-Ethiopia, for providing funding for this project. We would also like to thank Debre Berhan University, Asrat Woldeyes Health Science Campus for providing guidance and ethical standards for the implementation of the project. We also want to express our gratitude to all of our department employees and colleagues for their insightful advice and remarks.

# Disclosure of potential conflicts of interest

The authors declare that there are no competing interests or financial relationships that could be perceived to influence the conduct, analysis, or reporting of this research. Although this study received funding support from IPHC-E, the funder had no role in the study design, data collection and analysis, decision to publish, or preparation of the manuscript.

# Table of contents

Contents

[Investigators Affiliation	I](#_Toc215562711)

[Acknowledgment	II](#_Toc215562712)

[Disclosure of potential conflicts of interest	III](#_Toc215562713)

[Table of contents	IV](#_Toc215562714)

[List of tables	V](#_Toc215562715)

[List of figures	VI](#_Toc215562716)

[Abbreviations and acronyms	VII](#_Toc215562717)

[Summary	VIII](#_Toc215562718)

[1.	Introduction	1](#_Toc215562719)

[1.1.	Background	1](#_Toc215562720)

[1.2.	Problem statement	2](#_Toc215562721)

[1.3.	Significance of the study	3](#_Toc215562722)

[2.	Literature review	3](#_Toc215562723)

[3.	Project goal objective	5](#_Toc215562724)

[3.1.	General objective	5](#_Toc215562725)

[3.2.	Specific objectives	5](#_Toc215562726)

[3.3.	Research questions:	5](#_Toc215562727)

[3.4.	Hypothesis:	5](#_Toc215562728)

[4.	Methodology	6](#_Toc215562729)

[4.1.	Study design, setting, and population	6](#_Toc215562730)

[4.2.	Inclusion and exclusion criteria	6](#_Toc215562731)

[4.3.	Intervention	7](#_Toc215562732)

[4.4.	Control group	9](#_Toc215562733)

[4.5.	Clinical outcome	10](#_Toc215562734)

[4.6.	Operational definition	10](#_Toc215562735)

[4.7.	Sample size	11](#_Toc215562736)

[4.8.	Study participant’s selection procedure	11](#_Toc215562737)

[4.9.	Data collection tool and procedure	12](#_Toc215562738)

[4.10.	Data quality control	13](#_Toc215562739)

[4.11.	Data processing and analysis plan	14](#_Toc215562740)

[4.12.	Clinical significance	14](#_Toc215562741)

[4.13.	Ethical approval	14](#_Toc215562742)

[4.14.	Dissemination plan	15](#_Toc215562743)

[5.	Work plan	16](#_Toc215562744)

[6.	Budget	17](#_Toc215562745)

[7.	Reference	18](#_Toc215562746)

[Annex	21](#_Toc215562747)

[Information sheet	21](#_Toc215562748)

[Informed consent form	23](#_Toc215562749)

[Questionnaire	24](#_Toc215562750)

# List of tables

[Table 1: Components of maternal early warning scores (MEWS) criteria for prediction of Severe Maternal Morbidity, Ethiopia, 2025.	8](#_Toc200183724)

[Table 2: A schedule showing proposed key activities and time frame for assessing Maternal Early Warning System (MEWS) Model for predicting severe maternal morbidity and reducing maternal mortality in Ethiopia, 2025.	15](#_Toc200183725)

[Table 3: Budget breakdown to assess Maternal Early Warning System (MEWS) Model to predict severe maternal morbidity and reducing maternal mortality in Ethiopia, 2025.	16](#_Toc200183726)

# List of figures

[Figure 1: Maternal Early Warning Score (MEWS) Escalation Protocol for evaluating the effectiveness of the MEWS Model in predicting severe maternal morbidity and reducing maternal mortality in Ethiopia, 2025.	9](#_Toc200183731)

[Figure 2: Flow diagram showing selection of study participants to assess Maternal Early Warning System (MEWS) Model for predicting severe maternal morbidity and reducing maternal mortality in Ethiopia, 2025.	12](#_Toc200183732)

# Abbreviations and acronyms

ANC: Antenatal Care

AWHSC: Asrat Woldeyes Health Science Campus

DBU: Debre Berhan University

EDHS: Ethiopian Demographic and Health Survey

IPHC-E: International Institute for Primary Health Care-Ethiopia

LMICs: Low- and Middle-Income Countries

MEWS: Maternal Early Warning System

NGO: Non-governmental Organization

SDG: Sustainable Development Goal

SPSS: Statistical Package for Social Sciences

WHO: World Health Organization

# Summary

**Background:** Reducing maternal mortality remains a key indicator of healthcare quality, with severe maternal complications being the primary causes of death, despite being largely preventable. The maternal early warning system (MEWS) is the single most reliable response tool that detects changes in the clinical conditions and used to improve maternal and neonatal outcomes in obstetrics patients. MEWS use combined clinical observations to predict increased risk of deterioration and alert health workers to institute actions likely to improve outcomes. However, it was not applied consistently among inpatients at the bedside and there is limited evidence of its effectiveness.

**Objective:** To implement the Maternal Early Warning System (MEWS) for monitoring pregnant and postpartum inpatients and assess its effectiveness in predicting severe maternal morbidity and reducing maternal mortality as an alternative to standard clinical monitoring (vital sign sheet) in Public Hospitals of North Shewa Zone, Ethiopia.

**Method:** A parallel, quasi-experimental study design will be implemented among 1138 obstetric inpatients from May to September 2025 at four hospitals. The intervention group (n=569) will be followed using the MEWS chart and the parameters are systolic blood pressure, diastolic blood pressure, temperature, heart rate, respiratory rate, oxygen saturation, urine output, consciousness level, and pain assessment, and for postpartum women; vaginal bleeding, uterine contraction, and perineal tear. Each parameter will be assessed and categorized as yellow for moderate abnormalities, or red for severe derangements. for red, an obstetrician will be communicated to examine and decide for the patient. The control group will receive the standard clinical monitoring using the existing vital sign sheet (n=569). A multivariate generalized estimating equation (GEE) model with Poisson regression will be used to compare the outcomes and estimate adjusted risk ratios (aRR) with 95% confidence intervals. A statistically significant level will be declared at a p-value of <0.05.

**Keywords:** MEWS, Maternal deterioration, Maternal morbidity, Maternal mortality, Ethiopia

# 1. Introduction

## 1.1. Background

Despite a 34% global decline in maternal mortality from 2000 to 2020, 287,000 women died in 2020, with 95% of deaths occurring in low-income countries, including Ethiopia (1). In Ethiopia, the maternal mortality ratio dropped from 871 per 100,000 live births in 2000 to 267 in 2020 (2), yet it remains significantly above the SDG target of 70 per 100,000 (3). Maternal deaths represent only the “tip of the iceberg,” as the World Health Organization (WHO) estimates that for each maternal death, approximately 20 to 30 women experience maternal morbidity (4). Nearly 75% of maternal deaths result from five main causes: severe bleeding, infections, high blood pressure, complications during delivery, and unsafe abortion, most of which are preventable through early detection and timely intervention of complications (4). A medical research indicates that about 84% of maternal deaths are preventable with timely and appropriate care (5). But, delays in recognizing and managing complications remain a major challenge. Evidence from high-income countries shows that implementing the Maternal Early Warning System (MEWS) improves early detection and intervention, reducing severe maternal complications and mortality (6).

The MEWS is a structured approach that helps healthcare providers systematically monitor key physiological parameters, including the vital signs, oxygen saturation, urine output, consciousness level, pain assessment, uterine contraction, vaginal bleeding, and perineal tear to detect early signs of deterioration in pregnant and postpartum women (6). The MEWS is highly effective and excels at early identification of critically ill women (7). MEWS facilitates early recognition, diagnosis, and treatment of critical illnesses, enhancing maternal safety and reducing maternal morbidity and mortality (8). In Finland, the **Maternal Early Warning System** demonstrated high sensitivity in detecting the leading causes of maternal mortality, i.e., pre-eclampsia, sepsis, and postpartum hemorrhage (9). Similarly, in Indonesia, the implementation of MEWS improved the frequency of patient monitoring and facilitated timely clinical interventions for abnormal observations (10). In Nigeria, MEWS assisted healthcare providers in managing work demands, while also making it easier to detect and manage deteriorating patients (6).

## 1.2. Problem statement

Pregnancy and childbirth complications pose significant health risks for women and often lead to lasting physical and psychological effects. Maternal morbidity and mortality in Ethiopia is a complex and multifaceted issue influenced by numerous contributing factors. One of this factor is related to suboptimal maternal health service delivery (11). This is particularly critical because, even when women overcome the challenges of seeking and reaching care, poor evidence-based service delivery can still lead to severe complications or death. Addressing this gap requires strengthening health systems, improving training and supervision, and implementing quality improvement initiatives (12).

Despite ongoing efforts, Ethiopia continues to face high rates of maternal morbidity and mortality, much of which is preventable with timely and appropriate care. Current maternal monitoring systems in many healthcare facilities are often reactive rather than proactive, leading to delays in recognizing and managing obstetric complications. Current systems often fail to consistently identify women at risk until complications become life-threatening. The Maternal Early Warning System (MEWS) is a clinical tool designed to identify early signs of maternal deterioration through the systematic monitoring of physiological parameters. Implementing MEWS can enable timely interventions, prevent progression to severe complications, and ultimately reduce maternal deaths.

The MEWS was designed to account for the normal physiological changes of pregnancy. The intention of the MEWS is to improve recognition of pregnant women at risk of clinical deterioration and facilitate early intervention. Triggers in the MEWS system theoretically lead to earlier recognition of conditions that can contribute to maternal morbidity and mortality, including cardiovascular disease, sepsis, thromboembolic disease, hemorrhage and pre-eclampsia.1 The MEWS is intended to guide clinical judgement, and not replace it (13).

In addition to identifying patients at risk, MEWS also improve the reliability of taking and recording a full set of vital signs (14), e.g., respiratory rate is often not measured or recorded. Respiratory rate is one of the more sensitive parameters when trying to identify patients at risk of deterioration. A MEWS facilitates communication and provides an expectation and auditable standard for response by the medical team to deterioration in a pregnant woman’s physiology (13).

A number of international studies support the use of maternal early warning tools to address maternal complications. However, evidence on their effectiveness in reducing maternal morbidity and mortality within the African context remains limited. Similarly, the integration and adaptation of such tools into the Ethiopian maternal healthcare system remain limited, and their impact on maternal health outcomes has not been thoroughly assessed. Despite the persistently high maternal mortality rates in Ethiopia, there is currently no evidence on the effectiveness of the MEWS model in Ethiopian hospital settings. Thus, the study aims to assess the effectiveness of the MEWS model in predicting severe maternal morbidity and mortality in selected hospitals of North Shewa Zone, Amhara region, Ethiopia. The primary goal is to facilitate the early detection and management of patients showing signs of clinical deterioration and to generate evidence-based recommendations for scaling up and integrating the MEWS as a standard maternal health monitoring system.

## 1.3. Significance of the study

The MEWS is a commonly used example of a bedside screening tool that enables tracking of physiological parameters, and when a predefined threshold is reached, triggers bedside assessment by a healthcare professional. This implementation research will contribute to the body of evidence on the effectiveness of MEWS in low-resource settings. By piloting and evaluating the MEWS model in Ethiopia, the study aims to strengthen clinical decision-making, improve the quality of maternal care, and reduce delays in recognizing and managing severe maternal complications. The findings will inform policy and practice and improve maternal care quality, potentially leading to the national scale-up of MEWS as part of the maternal health strategy. Ultimately, the study supports Ethiopia’s efforts to achieve Sustainable Development Goal 3.1 by reducing maternal mortality and enhancing maternal health systems.

# 2. Literature review

The MEWS is designed to reduce delays in identifying severe maternal morbidities such as hemorrhage, preeclampsia, and sepsis, conditions that are among the leading causes of preventable maternal mortality (15). MEWS operates by prompting timely clinical interventions when physiological parameters exceed set thresholds.

Several studies have demonstrated the effectiveness of MEWS in improving maternal outcomes. A prospective study in London reported that 30% of patients triggered the MEWS criteria, and 13% developed morbidities including hemorrhage (43%), hypertensive disorders of pregnancy (31%), and suspected infections (20%) (16). In Spain, a prospective observational study showed MEWS was triggered in 6.43% of patients, with abnormal systolic blood pressure being the most common indicator (42.7%). Notably, 14.7% of these patients were diagnosed with potentially fatal disorders, and a significant association was found between MEWS activation and the presence of a potentially fatal disorder (17).

Similarly, findings from China revealed that women with severe obstetric complications had significantly higher MEWS scores compared to those without complications. Additionally, critically ill women in this study had shorter gestational ages and longer hospital stays (7). A study in Finland further confirmed that MEWS had high sensitivity in detecting leading causes of maternal mortality such as preeclampsia, sepsis, and postpartum hemorrhage. Specific red trigger indicators, such as systolic and diastolic blood pressure were independently associated with pre-eclampsia; systolic blood pressure and heart rate were associated with postpartum hemorrhage; and elevated heart rate was related to infection (9).

Implementation studies from Indonesia and Nigeria highlighted improvements in clinical practice following the adoption of MEWS. In Indonesia, the system enhanced the frequency of vital sign monitoring and facilitated timely responses to clinical deterioration (10). Similarly, in Nigeria, MEWS enhanced the regular monitoring of patients, supported healthcare providers in managing their workload, and facilitated the early detection and management of clinical deterioration (6).

Supporting these findings, a systematic review concluded that MEWS improves routine vital sign observation, reduces delays in clinical responses to abnormal signs, and can potentially lower the severity of maternal morbidity (8). However, not all findings were universally positive. A retrospective study in Florida, USA, reported that while MEWS was associated with earlier detection, it was linked to increased time before ICU admission and did not result in improved patient outcomes (18). This highlights the need for further research on implementation fidelity and contextual adaptation.

# 3. Project goal objective

## 3.1. General objective

To assess the effectiveness of the **Maternal Early Warning System (MEWS)** model for predicting severe maternal morbidity and reducing maternal mortality in North Shewa Zone, Ethiopia, 2025.

## 3.2. Specific objectives

1. To assess the effectiveness of the MEWS in predicting the number of patients triggered for potential severe maternal morbidity (SMM) and in reducing maternal mortality, compared to standard clinical monitoring.
2. To compare the rates of intensive care unit admissions, emergency surgical interventions, length of hospital stay, readmissions, time to diagnose severe maternal morbidity (SMM), time to clinical interventions, and the frequency of maternal monitoring.

## 3.3. Research questions:

1. Does the implementation of MEWS effective in predicting the number of patients triggered for potential severe maternal morbidity and in reducing maternal mortality?
2. Does the implementation of MEWS reduce ICU admission rates, emergency surgical interventions, length of stay, readmissions, shorten the time to diagnose SMM, decrease the time to clinical interventions, and increase the frequency of maternal monitoring?

## 3.4. ****Hypothesis:****

1. The MEWS is effective in predicting the number of patients triggered for potential severe maternal morbidity and in reducing maternal mortality.
2. The MEWS decreases ICU admissions, emergency surgical interventions, length of stay, readmissions, time to diagnose SMM, time to interventions, and increases the frequency of monitoring.

# 4. Methodology

## 4.1. Study design, setting, and population

A parallel, quasi-experimental study design will be implemented from May to September 2025 in North Shewa Zone, Amhara Region, Ethiopia. The Zone is bordered by the Oromia Region to the south and west, South Wollo to the north, and the Afar Region to the east. Debre Berhan is the capital city of the North Shewa Zone and is located about 130 kilometers northeast of Addis Ababa on the Ethiopian highway. The total population of the zone is estimated at 2,429,108, including 1,203,366 females (19). There are eleven public hospitals in the zone; 3 General hospitals (Mehal Meda, Alem-ketema Enat, and Debre Berhan University Hakim Gizaw Hospital), 7 primary hospitals, and 1 comprehensive Specialized hospital offering complete emergency obstetric care.

The study will be conducted in four selected hospitals: Debre Berhan University (DBU) Hakim Gizaw Hospital, Debre Berhan Comprehensive Specialized Hospital (CSH), Alem Ketema (Enat) Hospital, and Mehal Meda Hospital. DBU Hakim Gizaw Hospital and Debre Berhan CSH are both situated in Debre Berhan Town, within Basona Werana District. Mehal Meda Hospital is located in Mehal Meda Town, Menz Gera Midir District, while Alem Ketema (Enat) Hospital is located in Alem Ketema Town, Merhabete District.

## 4.2. Inclusion and exclusion criteria

Pregnant and postpartum women admitted to inpatient wards for complications developing antepartum (high-risk women), intrapartum, or postpartum care services (up to 42 days postpartum) will be included in the study. Women presenting with abortion-related complications or seeking safe abortion care, those who have undergone obstetric or gynecologic surgery, and women in labor with complications such as obstructed labor, sepsis, pre-eclampsia, anemia, HELLP syndrome, pulmonary embolism, hemorrhage, chorioamnionitis, pulmonary edema, or cardiac disease will be included. However, women in normal labor, women who died with accidental event, and those transferred directly to the intensive care unit without passing through inpatient wards will be excluded.

## 4.3. Intervention

The intervention group will utilize a statistically developed and validated maternal early warning system (MEWS) chart, the details of which have been published elsewhere (17,20). This chart will replace the standard vital signs sheet for all recruited participants at the intervention site. The MEWS chart will be designed as a simple, abnormal-observation-based recording tool, categorizing vital parameters as “Green” for no abnormalities, “Yellow” for moderate abnormalities, or “Red” for severe derangements, to facilitate the early detection of clinical deterioration.

- 🟩 **Green (Normal)**: No immediate concern
- 🟨 **Yellow Alert (Moderate abnormalities)**: Requires closer observation
- 🟥 **Red Alert (Severe derangements)**: Immediate clinical attention required

The MEWS criteria include the following twelve key clinical parameters (Table 1). If all parameters remain normal, routine monitoring continues as per standard practice. A patient is considered triggered for further assessment if they receive **one red score or two yellow** **scores.** In such cases, the midwife, GP, or accompanying healthcare provider will confirm observations, conduct a thorough history and examination, increase monitoring frequency, and re-evaluate the patient within 30 minutes. Additional actions include administering intravenous fluids, providing oxygen at 10 L/min if needed, providing antihypertensive medication, reviewing the observation and drug prescription charts, and, if the patient is pregnant, ensuring appropriate positioning such as tilting. If the patient stabilizes and returns to reassuring levels, routine monitoring continues. However, if the red scores persist or rise upon re-evaluation, immediate consultation with a senior obstetrician is required and patients will be reviewed within 60 minutes. Based on evaluation and diagnosis, emergency intervention, urgent referral, or ICU transfer will be initiated. If more than two yellow alerts or two or more red alerts are identified, the patient requires immediate review by an obstetrician. Reassess in 15 minutes, and start continuous monitoring. If the obstetrician is unable to resolve the situation, an anesthesiologist will be called.

Table 1: Components of maternal early warning scores (MEWS) criteria for prediction of Severe Maternal Morbidity, Ethiopia, 2025.

| **Physiologic parameters** | **Normal values** | **Yellow alert** | **Red alert** |
| --- | --- | --- | --- |
| Systolic blood pressure | 100 – 150 mmhg | 90 – 100 OR 150 – 160 | <90 OR ≥160 |
| Diastolic blood pressure | 50 – 89 mmhg | 90 – 110 | ≥110 |
| Temperature | 36 – 37.9 ◦C | 35 – 36 ◦C | ≤35 ◦C OR ≥38 |
| Heart rate | 50 – 99 BMP | 40 – 50 OR 100 – 130 | <40 OR >130 |
| Respiratory rate | 10 – 20 BMP | 21 – 30 | <10 OR >30 |
| Oxygen saturation | 95 – 100% | 90 – 95% | <90% |
| Urine outputs | ≥30 ml per hour | – | <35 ml/hour for 2 hours |
| Consciousness level | Alert | Responds to voice | To pain or unresponsive |
| Pain score | None to mild pain [0 – 3 pain score] | Intermittent pain at rest, moderate pain with movement [4 – 7 pain score] | Persistent pain at rest, severe pain with movement [8 – 10 pain score] |
| **For Postpartum or Post CS Women** | | | |
| Vaginal bleeding | No bleeding | Mild to moderate, i.e., spotting/light bleeding | **Active**, i.e., soaking a pad in <1 hour |
| Uterine contraction | Well contracted uterus | Soft but responsive to massage | Boggy, poorly/ non-contracted uterus |
| Perineal tear | No tear | 1^st^ and 2^nd^ degree tear | 3^rd^ and 4^th^ degree tear |

**Complete a full set of MEWS observations**

**≥ 3 yellow alerts or ≥ 2 red alerts:** immediate review by obstetrician and repeat observations in 15 minutes or continuous monitoring.

**2 yellow alerts or 1 red alert:** repeat observations in 30 minutes and if persists or worsens, consult the obstetrician.

**1 yellow alert:** repeat observations in 30 minutes and continue monitoring as per standard practice.

**If all parameters remain normal**, routine monitoring continues as per standard practice.

Figure 1: Maternal Early Warning Score (MEWS) Escalation Protocol for evaluating the effectiveness of the MEWS Model in predicting severe maternal morbidity and reducing maternal mortality in Ethiopia, 2025.

## 4.4. Control group

The control hospitals will continue with their existing/standard clinical monitoring practices. This involves monitoring and recording temperature, pulse, blood pressure, and respiratory rate on vital sign sheets. The MEWS chart will be used for the intervention hospitals, while the existing vital signs chart will be utilized for the control hospitals.

## 4.5. Clinical outcome

The primary clinical outcome is the **number of patients triggered by MEWS** for potential severe maternal morbidity and a reduction in maternal mortality. Secondary outcomes include a decrease in intensive care unit (ICU) admissions, emergency surgical interventions, length of stay, time to diagnosis, time to intervention, increased frequency of MEWS recording, and increased adherence to maternal monitoring through the use of the MEWS chart.

We hypothesized that, the MEWS helps reduce ICU admissions and emergency surgical interventions by enabling early detection and timely response to clinical deterioration in pregnant and postpartum women. It monitors vital signs and identify early warning signs of complications such as hemorrhage, sepsis, and preeclampsia. When abnormal values are detected, MEWS prompts a standardized clinical response, ensuring rapid assessment and intervention. This proactive approach prevents conditions from worsening, reducing the likelihood of critical illness, intensive care needs, and emergency procedures. Studies have shown that MEWS improves the frequency of vital sign monitoring, enhances team communication, and leads to earlier treatment, ultimately improving maternal outcomes. However, it may also increase as an unintended outcome of earlier detection of potential severe maternal deterioration, which could lead to more frequent early transfers and increased ICU admissions (21).

**ICU Admission Data:** Information on ICU admission will be extracted from patient records, including: whether the patient was admitted to the ICU (Yes/No), indication for ICU admission, and timing of admission relative to diagnosis of complications. Baseline clinical characteristics, type and timing of interventions, vital signs or MEWS scores, and outcomes will also be collected. The **proportion of ICU admissions** in the intervention group will be compared to the proportion in the control group.

## 4.6. Operational definition

**Triggering on MEOWS chart:** A trigger is defined as a single markedly abnormal observation (red trigger) or a combination of two simultaneous mildly abnormal observations (two yellow triggers) (22).

**Severe maternal morbidity:** is a clinical condition or diseases that can threaten a woman’s life during pregnancy and labour and after termination of pregnancy (23). It will be assessed using the Centers for Disease Control and Prevention (CDC) International Disease classification indicators (21,24). It includes hemorrhage, sepsis, pre-eclampsia, eclampsia, shock, acute renal failure, cardiovascular disorder (stroke), heart failure, severe anemia, pulmonary edema, hysterectomy, and thrombotic embolism.

**Composite maternal morbidity:** refers to the occurrence of one or more severe complications during pregnancy, childbirth, or within 42 days postpartum, including secondary outcomes (21).

**Maternal death:** death of a woman while pregnant or within 42 days of termination of pregnancy, irrespective of the duration and the site of the pregnancy, from any cause related to or aggravated by the pregnancy or its management, but not from accidental or incidental causes (23).

**Emergency surgical interventions:** include emergency cesarean section, **surgical repair of uterine rupture**, hysterectomy, **laparotomy, uterine artery ligation, compression sutures (e.g., b-lynch sutures)**, **repair of perineal or vaginal tears**, drainage of pelvic abscess or puerperal sepsis, and Wound Re-exploration for surgical site infection (SSI).

## 4.7. Sample size

There was no comparable baseline study in Ethiopia. However, we know that the incidence of severe maternal morbidity (SMM) in our setting is 14.3% (25). We hypothesize that the MEWS intervention would increase patients triggered for potential SMM by 21% (16). To detect such a difference at a significance level of 0.05 with 80% power, each arm would require a minimum of 542 cases. Adding 5% for losses to follow-up, the study will require a total of 1138 samples; 569 in the intervention and 569 in the control group.

## 4.8. Study participant’s selection procedure

Four hospitals were purposely selected. Debre Berhan University Hakim Gizaw Hospital and Debre Berhan Comprehensive Specialized Hospital (CSH) will serve as the intervention hospitals while Enat Hospital and Mehalmeda Hospital will be used as the control hospitals. This is because the hospitals are comparable in terms of the services they offer, the presence of obstetricians, the availability of intensive care unit, and their status as government health facilities. Then, the determined samples will be proportionately distributed to each hospital based on the hospital's six-month high-risk and postnatal care contact. Lastly, to find the required study participants, a systematic random sampling procedure with a sampling interval of two will be employed. Each hospital will use a lottery to select the first participant. Then, until the necessary sample size is attained, women who fulfill the inclusion criteria and are willing to take part in the study will be chosen. Geographical separation between the hospitals aids in preventing information contamination. We plan to speak with every possibly eligible woman at every hospital about the study and our care interactions.

**SyRS**

**PPS**

**North Shewa Zone**

**Hospitals**

Debre Berhan CSH: 623

Hakim Gizaw H:

581

Mehalmeda H:

469

275

278

294

**1138 (569:569)**

Enat Hospital:

492

291

**:** Ratio of intervention to control group	PPS: Probability proportional to size	SyRS: Systematic random sampling

Figure 2: Flow diagram showing selection of study participants to assess Maternal Early Warning System (MEWS) Model for predicting severe maternal morbidity and reducing maternal mortality in Ethiopia, 2025.

## 4.9. Data collection tool and procedure

Midwives, interns, or GPs will be made aware of the importance of accurately and completely charting patients’ parameters and the need for mandatory callout and review by an obstetrician following a trigger. Compliance and completeness of the MEWS chart will also be audited. However, to minimize observer bias, they will not be briefed about the proposed study. The women will be followed from enrollment in the study to discharge from the hospital. In both intervention and control hospitals, monitoring will involve recording parameters every 6 hours, or more frequently if indicated by the managing clinician. Frequency of observations is determined by risk status, diagnosis, reason for admission, and initial observations on admission. An individual plan of care which specify the frequency of physiological observations will be decided by the obstetricians. Midwives will monitor patients using the MEWS chart for two hours after childbirth or cesarean delivery, either in the delivery room or the post-anesthesia care unit (PACU), and will continue monitoring in the postnatal or post-cesarean section ward according to the physician order or protocol. The data collection tool was developed based on a review of various instruments used in previous studies (6,13,17,20,21). Midwives will conduct all MEWS assessments during inpatient care, monitoring clinical conditions, identifying triggers or complications, and ensuring timely responses. All findings will be thoroughly documented in the woman's medical record. Information will be obtained through medical record reviews and face-to-face interviews with the women. An independent data collector, blinded to group allocation, will immediately extract maternal outcome information and MEWS-related data from the medical records within 24 hours of discharge.

## 4.10. Data quality control

Back-and-forth questionnaire translation, from English to Amharic and vice versa, will be performed by reproductive health experts to check the consistency of the questions with the original meaning. A pre-test involving 5% (28 from the intervention group and 28 from the control group) of the sample size will be conducted at Kemisse Hospital to evaluate the validity and consistency of the tool. Based on the findings, appropriate modifications will be made as needed. Twelve BSc midwives (data collectors) and six MSc midwives (supervisors) will receive training on the proper use of the MEWS chart, standardized clinical definitions, data collection tool, and ethical considerations. During the data collection process, on-site supervision will be done. The supervisors and investigators will closely follow the completeness, coherence, and clarity of the data across the entire data collection period. To lessen the Hawthorne effect, we will establish a good rapport and spend more time with study participants, as well as, the clinical outcome data will be blinded for the healthcare providers employed in the maternity care units.

## 4.11. Data processing and analysis plan

The data will be collected using Kobo Toolbox version **2.025.02h** and analyzed using IBM SPSS version 25.0 statistical software. Descriptive statistics, i.e., mean, standard deviation, frequencies, and percentages will be used to analyze the baseline characteristics and clinical outcomes of study participants. The findings will be presented using narratives, figures, and tables. Proportions will be compared using Pearson’s Chi-Square (χ2) test for categorical variables. For continuous variables, a comparison of means will be done using the Student’s t-tests for normally distributed data or Mann-Whitney U tests for non-normally distributed data. To evaluate the effect of the MEWS intervention, a multivariate generalized estimating equation (GEE) model with Poisson regression will be used to compare the outcomes and estimate adjusted risk ratios (aRR) with 95% confidence intervals. A statistically significant level will be declared at a p-value of <0.05.

## 4.12. Clinical significance

MEWS are a valuable and effective bedside screening tools for identifying severe morbidity and mortality in obstetric patients. The initiative aims to enhance the early recognition of changes in maternal physiologic signs and clinical conditions, leading to timely escalation and prompt evaluation. This approach aims to reduce preventable adverse outcomes, including maternal deaths. MEWS may improve the frequency of routine monitoring and reduce the time between patient deterioration and corrective clinical action. These improvements can potentially enhance the quality of care for pregnant and postpartum women, thereby reducing the risk of adverse obstetric outcomes. The MEWS model has been validated and proven effective in predicting and reducing severe morbidity and mortality in low-resource settings (20).

## 4.13. Ethical approval

The project will be reviewed and ethical clearance will be obtained from the Institutional Review Board of Debre Berhan University (DBU). A formal support letter will be written to the study hospitals and a permission letter will be obtained from the hospital administrations/ CCD. The data collectors will explain the objective, potential risks, and benefits to the study participants. Participants will be informed that the participation will be voluntary and they can also withdraw from participating in the study at any time. Informed signed consent will be obtained from each participant. Those who cannot read and write will be asked to thumbprint the consent form after the information is read. Confidentiality and anonymity will be assured and the information will be used only for research purposes.

## 4.14. Dissemination plan

The result of the study will be submitted to the funding organization, the International Institute for Primary Health Care-Ethiopia (IPHC-E). Further, the information will be disseminated to the study hospitals, zonal health department, DBU, Asrat Woldeyes Health Science Campus Research, Community Service, and CBE Director, and for concerned stakeholders (NGOs) working on Maternal and Child Health in the study area. Besides, it will be presented at seminars, symposiums, or workshops, and it will be published in one of the international peer-reviewed journals.

# 5. Work plan

Table 2: A schedule showing proposed key activities and time frame for assessing Maternal Early Warning System (MEWS) Model for predicting severe maternal morbidity and reducing maternal mortality in Ethiopia, 2025.

| **S. No** | **Activities** | **Responsible body** | **Time frame** |
| --- | --- | --- | --- |
| 1. | Proposal and tool development | Investigators | April 10 – 19/2025 |
| 2. | MEWS Chart development | Investigators and senior Obstetrician and Gynecology specialists | April 20 – 21/2025 |
| 3. | Ethical Approval | The principal investigator and DBU IRB | April 22 – 24/2025 |
| 4. | Fulfilling necessary materials | Investigators and DBU finance and recruitment | April 25 – 29/2025 |
| 5. | Recruitment and training | Investigators | April 30 – May 2/2025 |
| 6. | Pre-testing | Investigators | May 3 – 4/2025 |
| 7. | Implementation of the project, supervision, and follow-up. | Investigators, data collectors, and supervisors | May 5 – September 31/2025 |
| 8. | Data processing, analysis, and interpretations | Investigators, data collectors, and supervisors | October 2025 |
| 9. | Provide a final report | Investigators | October 2025 |
| 10. | Dissemination and Journal submission | Investigators | November 2025 |

# 6. Budget

Table 3: Budget breakdown to assess Maternal Early Warning System (MEWS) Model to predict severe maternal morbidity and reducing maternal mortality in Ethiopia, 2025.

| **S. No** | **Activities** | **Measurement** | **Quantity** | **No of days** | **Unit cost in USD** | **Total cost** |
| --- | --- | --- | --- | --- | --- | --- |
| 1. | Proposal and tool development | Person | 5 | 10 | 6 | 300 |
| 2. | MEWS Chart development | Person | 5 | 2 | 4 | 40 |
| 3. | Training, workshop, and meeting. | Person | 25 | 3 | 20 | 1,500 |
| 4. | Pre-testing | Person | 6 | 2 | 5 | 60 |
| 5. | Follow ups and data collection. | Person | 12 | 4 months | 100 | 4,800 |
| 6. | Supervision | Person | 6 | 2 months | 150 | 1,800 |
| 7. | Analysis, write up, and dissemination | Person | 5 | 5 | 60 | 1,500 |
|  |  | **Total** | | | | **10,000 $** |

USD ($): US Dollar

# 7. Reference

1.	Maternal mortality [Internet]. [cited 2025 Mar 15]. Available from: https://www.who.int/news-room/fact-sheets/detail/maternal-mortality

2.	Organization WH. Trends in maternal mortality 2000 to 2020: estimates by WHO, UNICEF, UNFPA, World Bank Group and UNDESA/Population Division. World Health Organization; 2023.

3.	Bhowmik J, Biswas RK, Ananna N. Women’s education and coverage of skilled birth attendance: An assessment of Sustainable Development Goal 3.1 in the South and Southeast Asian Region. PLoS One. 2020;15(4):e0231489.

4.	Organization WH. Maternal morbidity and well-being. Maternal Health Unit. Retrieved January; 2024.

5.	Katella K. Maternal mortality is on the rise: 8 things to know. Yale Med. 2023;22.

6.	Umar A, Ibrahim S, Liman I, Chama C, Ijaiya M, Mathai M, et al. Implementation and Evaluation of Obstetric Early Warning Systems in tertiary care hospitals in Nigeria. PLOS Glob Public Heal. 2022;2(7):e0000225.

7.	Xu Y, Zhu S, Song H, Lian X, Zeng M, He J, et al. A new modified obstetric early warning score for prognostication of severe maternal morbidity. BMC Pregnancy Childbirth. 2022;22(1):901.

8.	Umar A, Ameh CA, Muriithi F, Mathai M. Early warning systems in obstetrics: A systematic literature review. PLoS One. 2019;14(5):e0217864.

9.	Hannola K, Hoppu S, Mennander S, Huhtala H, Laivuori H, Tihtonen K. Obstetric early warning system to predict maternal morbidity of pre-eclampsia, postpartum hemorrhage and infection after birth in high-risk women: a prospective cohort study. Midwifery. 2021;99:103015.

10.	Sasmito P, Pribadi DRA, Juliastuti D, Pamungkas RA, Setyowati TA, Jumintono J, et al. Early warning system in obstetrics, MEOWS: A systematic literature review. Bali Med J. 2024;13(3):1094–103.

11.	Oleribe OO, Momoh J, Uzochukwu BSC, Mbofana F, Adebiyi A, Barbera T, et al. Identifying key challenges facing healthcare systems in Africa and potential solutions. Int J Gen Med. 2019;395–403.

12.	Kruk ME, Gage AD, Arsenault C, Jordan K, Leslie HH, Roder-DeWan S, et al. High-quality health systems in the Sustainable Development Goals era: time for a revolution. Lancet Glob Heal. 2018;6(11):e1196–252.

13.	Nair S, Dockrell L, Mac Colgain S. Maternal early warning scores (MEWS). 2018;

14.	Hammond NE, Spooner AJ, Barnett AG, Corley A, Brown P, Fraser JF. The effect of implementing a modified early warning scoring (MEWS) system on the adequacy of vital sign documentation. Aust Crit Care. 2013;26(1):18–22.

15.	Chimwaza Y, Hunt A, Oliveira-Ciabati L, Bonnett L, Abalos E, Cuesta C, et al. Early warning systems for identifying severe maternal outcomes: findings from the WHO global maternal sepsis study. EClinicalMedicine. 2025;79.

16.	Singh S, McGlennan A, England A, Simons R. A validation study of the CEMACH recommended modified early obstetric warning system (MEOWS). Anaesthesia. 2012;67(1):12–8.

17.	Ibáñez-Lorente C, Casans-Francés R, Bellas-Cotán S, Muñoz-Alameda LE. Implementation of a maternal early warning system during early postpartum. A prospective observational study. PLoS One. 2021;16(6):e0252446.

18.	Polic A, Kramer E, Louis J. 983: Effects of implementing a maternal early warning system at a tertiary care center. Am J Obstet Gynecol. 2020;222(1):S611.

19.	CSA. Ethiopian 2022 Project Population Census. 2022;(July):13.

20.	Umar A, Manu A, Mathai M, Ameh C. Development and validation of an obstetric early warning system model for use in low resource settings. BMC Pregnancy Childbirth. 2020;20:1–9.

21.	Shields LE, Wiesner S, Klein C, Pelletreau B, Hedriana HL. Use of maternal early warning trigger tool reduces maternal morbidity. Am J Obstet Gynecol. 2016;214(4):527-e1.

22.	Radha R, Kokila S, Mukilvizhi S, Venkatesan R. Evaluation of the effectiveness of modified early obstetric warning system in a tertiary health care setup. Int J Sci STUDY. 2021;9(1):167–77.

23.	WHO. Evaluating the quality of care for severe pregnancy complications: the WHO near-miss approach for maternal health. 2011;

24.	Metcalfe A, Sheikh M, Hetherington E. Impact of the ICD-9-CM to ICD-10-CM transition on the incidence of severe maternal morbidity among delivery hospitalizations in the United States. Am J Obstet Gynecol. 2021;225(4):422-e1.

25.	Gebremariam TB, Demie TG, Derseh BT, Mruts KB. Trends of and Factors Associated with Maternal Near‐Miss in Selected Hospitals in North Shewa Zone, Central Ethiopia. J Pregnancy. 2022;2022(1):2023652.

# Annex

## Information sheet

**Introduction:** My name is­­_________________. This is an information sheet prepared to research the topic entitled “Maternal Early Warning System (MEWS) Model for predicting severe maternal morbidity and reducing maternal mortality in Ethiopia”. The principal investigator is Saba Desta, and the co-investigators are Mesfin Tadese, Dr. Solomon Hailemeskel, Dr. Getaneh Dejen, Chaltu Takele, and Dr. Lidya Asalefew, a lecturer at Debre Berhan University, Asrat Woldeyes Health Science Campus. You are randomly selected to participate in this study. Before you decide whether or not to participate, I would like you to explain the objective, risks, benefits, procedure, and what is expected of you.

**Objective**: To assess the effectiveness of Maternal Early Warning System (MEWS) Model for predicting severe maternal morbidity and reducing maternal mortality in North Shewa Zone Public Hospitals, Ethiopia, 2025.

**Procedure:** A formal support letter will be obtained from the Research and Community Service Corporate Director of the Asrat Woldeyes Health Science Campus. Upon signing the informed consent form, you will be monitored using the Maternal Early Warning System (MEWS) chart. Data collection will involve a face-to-face interview and a review of your clinical records. In addition, the data collector will closely follow your condition and progress throughout your hospital stay until discharge. After consent is obtained, the data collector will routinely document information related to your antenatal, labor and delivery, and postnatal care.

**Benefits:** For your participation in the study, no payment will be granted or has no special privilege to you. However, participating in the study and giving your information will have great input in the efforts made to reduce severe maternal morbidity and mortality and minimize its impact on community at large.

**Risks:** The study will be done simply by following your pregnancy, using the already prepared structured questions. The procedure doesn't bear any physical or psychological harm/trauma. Furthermore, you will not be forced to respond to the information you do not know and want to answer.

**Confidentiality**: All information you give will be strictly confidential and will be kept safe and secure. Your name will not appear anywhere on the questionnaire sheet and the information you are going to provide is used only for research purposes.

**Rights of participants:** You are completely free to take part or not in this study. You have the right to withdraw from the study at any time you want or abstain from responding to questions you don’t want to answer. You can ask any question which is not clear to you.

Would you like to participate in the study?

1. Yes (Take informed consent)
2. No (Say thank you)

## Informed consent form

I have been informed that the interview is to gather information regarding the “Effectiveness of Maternal Early Warning System (MEWS) Model for predicting severe maternal morbidity and reducing maternal mortality in Ethiopia”. The objective, procedure, benefits, and harms of the study have been read and explained to me in a language I understand. I further appreciate that taking part in this study and withdrawing from participating at any time without having a reason is purely voluntary. I also agreed about the confidentiality of the responses to be at a higher possible level. Therefore; I declare my voluntary consent to participate in this study with my initials (signature) as indicated below.

Participant’s signature _______________ Date _________________

Data collector: Name__________________

Signature ______________ Date________________

Supervisor: Name: ____________________Signature: ____________ Date___________

If you have any questions, you can contact the AWHSC, DBU IRB:

Administrative Building: 6^th^ floor

Office Number: F6-02

Tel: +251912372837

Email: [AWHSC-IRB@dbu.edu.et](mailto:AWHSC-IRB@dbu.edu.et)

**Thank you for your co-operation!**

## Questionnaire

Questionnaire code: _____________ Hospital: _________________ MRN: ______________

**Part-I: Socio-demographic characteristics**

| **S. No** | **Questions** | **Responses options** | **Skip** |
| --- | --- | --- | --- |
| 1. | What is your age | **________**Years |  |
| 2. | What is your place of residence? | 1. Urban 2. Rural |  |
| 3. | What is your marital status? | 1. Single 2. Married 3. Divorced 4. Widowed |  |
| 4. | What is your educational level? | 1. No formal education 2. Primary education 3. Secondary education 4. College and above |  |
| 5. | What is your occupation? | 1. Employed 2. Unemployed 3. Retired |  |
| 6. | How many minutes does it take to reach the hospital from your home? | _________ minutes. |  |
| 7. | What was type of transport did you use to reach the hospital? | 1. Ambulance 2. Public transport/ Taxi/ Bajaj 3. Private car 4. On foot |  |
| 8. | Do you have community-based health insurance (CBHI) | 1. Yes 2. No |  |
| 9. | How many children do you have? | ____________ |  |
| 10. | What is your family size? | ____________ |  |
| 11. | Were you referred from another health facility? | 1. Yes 2. No |  |
| 12. | What is your average monthly household income? | _____________ Birr. |  |

**Part II: Obstetric and reproductive health factors**

| **S. No** | **Questions** | **Responses options** | **Skip** | |
| --- | --- | --- | --- | --- |
| 1. | How many times have you been pregnant (Gravidity)? | _________ |  | |
| 2. | How many births have you had after 28 weeks including both alive and stillbirths (Parity)? | _________ |  | |
| 3. | Did you attend antenatal care (ANC) during this pregnancy? | 1. Yes 2. No | If “No” skip to Q6 | |
| 4. | If **Yes**, how many ANC visits did you attend during this pregnancy? | _________times |  | |
| 5. | At what week did you start ANC in this pregnancy? | _________ weeks |  | |
| 6. | Do you have history of Maternal obstetric complication in previous pregnancies? | 1. Yes 2. No | If “No” skip to Q8 | |
| 7. | If **Yes**, select all that apply (More than one answer is allowed) | 1. Pre-eclampsia 2. PROM 3. Hemorrhage 4. Sepsis 5. Anemia 6. ICU admission 7. GDM 8. Oligohydramnios 9. Others___________ |  | |
| 8. | Do you have any previous history of hospital admission related to pregnancy or childbirth complications? | 1. Yes 2. No | If “No” skip to Q10 | |
| 9. | If yes, what was the specific complication or reason for the admission? | 1. Pre-eclampsia 2. PROM 3. Antepartum hemorrhage 4. Sepsis 5. Anemia 6. Preterm labor 7. GDM 8. Oligohydramnios 9. Surgical site infection 10. Other specify _________ |  | |
| 10. | Do you have history of perinatal complication in previous pregnancy? | 1. Yes 2. No | If “No” skip to Q12 | |
| 11. | If **Yes**, select (More than one answer is allowed) | 1. Abortion 2. Stillbirth 3. Neonatal death 4. Fetal growth restriction 5. Low birth weight 6. Macrosomia 7. Prematurity 8. Congenital anomaly 9. Perinatal asphyxia 10. Meconium aspiration syndrome 11. Admission to NICU 12. Need for resuscitation | |  |
| 12. | Current pregnancy type | 1. Singleton 2. Twin | |  |
| 13. | Location of mother’s ward | 1. High risk (not in labor) 2. Labor 3. Postpartum/Post CS | |  |
| 14. | Do you have any known pre-existing medical illness? | 1. Yes 2. No | | If “No” skip to Q16 |
| 15. | If **Yes,** please specify the disease? (More than one answer is allowed) | 1. Hypertension 2. Diabetes 3. Cardiac disease 4. Kidney disease 5. Liver disease 6. HIV/AIDS 7. Others ___________ | |  |
| 16. | What was the interpregnancy interval (duration between the last date of birth and LNMP of this pregnancy) in months? | _________ months. |  | |
| 17. | What was the diagnosis at admission? | 1. Pre-eclampsia 2. PROM 3. Hemorrhage 4. Sepsis 5. Labor and delivery 6. Anemia 7. Gestational diabetes 8. Others specify_________ |  | |
| 18. | Gestational age at admission | ________ weeks. |  | |
| 19. | Gestational age at delivery | _________ weeks |  | |
| 20. | Was the current pregnancy planned? | 1. Yes 2. No |  | |
| 21. | Do you use alcohol or other drugs? | 1. Yes 2. No |  | |
| 22. | Was there any delay in receiving care at the facility? | 1. Yes 2. No |  | |
| 23. | Was there any experience of physical or emotional abuse during pregnancy? | 1. Yes 2. No |  | |

**Part III: MEWS triggering and monitoring**

| **S. No** | **Questions** | **Responses options** | **Skip** |
| --- | --- | --- | --- |
| 1. | **Type of monitoring used:** | 1. MEWS chart 2. Standard clinical monitoring (Vital sign sheet) | If “2” skip to Q17 |
| 2. | Was the MEWS chart filled completely? | 1. Yes 2. No |  |
| 3. | Did the patient have abnormal MEWS finding (≥1 red score or ≥2 yellow scores)? | 1. Yes 2. No | If No skip to Q17 |
| 4. | If Yes, what was the alert finding for a triggered patient? | 1. ≥2 yellow scores 2. 1 red score 3. ≥2 red scores |  |
| 5. | What was the alert finding (Sys BP, Dys BP, HR, O_2_ sat…) | _______________ |  |
| 6. | What was the **duration** from symptom onset to the MEWS trigger? | __________ hours |  |
| 7. | **What was the duration from admission to the first MEWS trigger? (in hours)** | __________ hours |  |
| 8. | What was the suspected complication based on the trigger? | 1. Pre-eclampsia 2. Eclampsia 3. Hypovolemia/hypotension 4. PPH 5. Sepsis 6. Anemia 7. Magnesium sulfate toxicity 8. Pulmonary edema 9. Pulmonary embolism 10. Chorioamnionitis 11. Surgical site infection 12. Other specify_________ |  |
| 9. | Was a physician consulted in response to the MEWS trigger? | 1. Yes 2. No | If “No” skip to Q12 |
| 10. | If **Yes**, what was the physician’s diagnosis after the consultation? | _______________ |  |
| 11. | What was the time interval between the MEWS trigger and the physician’s evaluation following consultation? | __________ minutes. |  |
| 12. | **Was clinical action taken in response to the MEWS trigger?** | 1. Yes 2. No | If “No” skip to Q15 |
| 13. | What type of action was taken in response to MEWS triggering? | 1. Continuous monitoring only 2. Repeat observations/ monitoring in 30 minutes. 3. Repeat observations/ monitoring in 15 minutes. 4. Blood transfusion 5. Resuscitation (O_2_ administration, IV fluid, and positioning) 6. Medication prescription 7. Emergency surgical intervention 8. Transfer to ICU 9. Referral to another facility 10. No action taken 11. Other specify__________ |  |
| 14. | If you select emergency surgical intervention, please specify? | 1. Emergency cesarean section 2. Hysterectomy 3. **Laparotomy** 4. **MVA (D and E/suction)** 5. **Surgical repair of uterine rupture** 6. **Uterine artery ligation** 7. **Compression sutures (e.g., b-lynch sutures)** 8. **Repair of perineal or vaginal tears** 9. Drainage of pelvic abscess or puerperal sepsis. 10. Wound Re-exploration for SSI 11. Other (specify): _______ |  |
| 15. | What was the duration from MEWS trigger (abnormal MEWS) to clinical intervention / action? (in minutes) | __________ minutes. |  |
| 16. | Was MEWS escalation protocol followed appropriately? | 1. Yes 2. No |  |
| 17. | Was the vital sign sheet filled completely? | 1. Yes 2. No |  |
| 18. | Were abnormal vital signs obtained? | 1. Yes 2. No | If No skip to Q30 |
| 19. | What was the abnormal vital sign finding (Sys BP, Dys BP, HR, RR, T^o^, O_2_ sat…) | _______________ |  |
| 20. | What was the duration from symptom onset to the abnormal vital sign record? | __________ hours |  |
| 21. | **What was the duration from admission to the abnormal vital sign? (in hours)** | __________ hours |  |
| 22. | What was the suspected complication based on the abnormal vital sign finding? | 1. Pre-eclampsia 2. Eclampsia 3. Hypovolemia/hypotension 4. Hemorrhage 5. Sepsis 6. Anemia 7. Magnesium sulfate toxicity 8. Pulmonary edema 9. Pulmonary embolism 10. Chorioamnionitis 11. Surgical site infection 12. Other specify_________ |  |
| 23. | Was a physician consulted in response to the abnormal vital sign? | 1. Yes 2. No | If “No” skip to Q26 |
| 24. | If **Yes**, what was the physician’s diagnosis after the consultation? | _______________ |  |
| 25. | What was the time interval between the abnormal vital sign and the physician’s evaluation following consultation? | __________ minutes. |  |
| 26. | **Was clinical action taken in response to the abnormal vital sign sheet trigger?** | 1. Yes 2. No | If “No” skip to 30 |
| 27. | What type of action was taken in response to abnormal vital sign trigger? | 1. Continuous monitoring only 2. Repeat observations/ monitoring in 30 minutes. 3. Repeat observations/ monitoring in 15 minutes. 4. Blood transfusion 5. Resuscitation (O_2_ administration, IV fluid, and positioning) 6. Medication prescription 7. Emergency surgical intervention 8. Transfer to ICU 9. Referral to another facility 10. No action taken 11. Other specify__________ |  |
| 28. | If you select emergency surgical intervention, please specify? | 1. Emergency cesarean section 2. Hysterectomy 3. **Laparotomy** 4. **MVA (D and E/suction)** 5. **Surgical repair of uterine rupture** 6. **Uterine artery ligation** 7. **Compression sutures (e.g., b-lynch sutures)** 8. **Repair of perineal or vaginal tears** 9. Drainage of pelvic abscess or puerperal sepsis. 10. Wound Re-exploration for SSI 11. Other (specify): _______ |  |
| 29. | What was the duration from the abnormal vital sign trigger to clinical intervention / action? (in minutes) | __________ minutes. |  |
| 30. | Number of ultrasound scans after admission? | ___________ |  |

**Part IV: Severe Maternal Morbidity and Mortality**

| **S. No** | **Questions** | **Responses options** | **Skip** |
| --- | --- | --- | --- |
| 1. | Did the women develop any form of Severe Maternal Morbidity (SMM) during her hospital stay after admission to the ward? | 1. Yes 2. No | If “No” skip to part V |
| 2. | If **Yes**, please specify**?** *(More than one answer is allowed)* | 1. Hemorrhage 2. Severe pre-eclampsia 3. Eclampsia 4. Sepsis 5. Anemia 6. Uterine rupture 7. Pulmonary edema 8. Respiratory distress 9. Need for blood transfusion 10. Admission to ICU 11. Hysterectomy 12. Organ failure 13. Maternal death 14. Other specify______ |  |
| 3. | When did the woman develop Severe Maternal Morbidity (SMM) after being admitted? | _________ hours. |  |
| 4. | What was the duration from MEWS trigger/ abnormal vital sign record to the diagnosis of SMM? | _________ hours. |  |
| 5. | Was the MEWS trigger/ abnormal vital sign record predictive of the SMM condition? | 1. Yes 2. No |  |

**Part V: Clinical Outcome Comparisons**

| **S. No** | **Questions** | **Responses options** | **Skip** |
| --- | --- | --- | --- |
| 1. | **Length of hospital stay (in days)** | **________** days |  |
| 2. | Was the patient readmitted for maternal complications after discharge? | 1. Yes 2. No |  |
| 3. | Was the patient admitted to the Intensive Care Unit (ICU)? | 1. Yes 2. No |  |
| 4. | **How frequently was the patient monitored either using the MEWS chart or vital sign sheet?** | 1. Every 4 hours 2. Every 6 hours 3. Once per shift 4. Irregularly 5. Not documented 6. Other specify___________ |  |
| 7. | Outcome at discharge: | 1. Stable, discharged home 2. Transferred to ICU 3. Referred to another facility 4. Maternal death 5. Discharged against medical advice 6. Lost to follow-up |  |

**Thank you very much!!!**
